# Structure of silent transcription intervals and noise characteristics of mammalian genes

**DOI:** 10.15252/msb.20156257

**Published:** 2015-07-27

**Authors:** Benjamin Zoller, Damien Nicolas, Nacho Molina, Felix Naef

**Affiliations:** The Institute of Bioengineering, School of Life Sciences, Ecole Polytechnique Fédérale de LausanneLausanne, Switzerland

**Keywords:** noise in mRNA counts, promoter cycle, single-cell time-lapse analysis, stochastic gene expression, transcriptional bursting

## Abstract

Mammalian transcription occurs stochastically in short bursts interspersed by silent intervals showing a refractory period. However, the underlying processes and consequences on fluctuations in gene products are poorly understood. Here, we use single allele time-lapse recordings in mouse cells to identify minimal models of promoter cycles, which inform on the number and durations of rate-limiting steps responsible for refractory periods. The structure of promoter cycles is gene specific and independent of genomic location. Typically, five rate-limiting steps underlie the silent periods of endogenous promoters, while minimal synthetic promoters exhibit only one. Strikingly, endogenous or synthetic promoters with TATA boxes show simplified two-state promoter cycles. Since transcriptional bursting constrains intrinsic noise depending on the number of promoter steps, this explains why TATA box genes display increased intrinsic noise genome-wide in mammals, as revealed by single-cell RNA-seq. These findings have implications for basic transcription biology and shed light on interpreting single-cell RNA-counting experiments.

## Introduction

Gene expression is intrinsically dynamic and varies greatly from cell to cell (Raj & van Oudenaarden, 2008). In isogenic cell populations, such variability arises naturally from randomness in the processes governing gene expression. Typically, low numbers of molecules are involved in transcription, leading to unavoidable stochasticity in both mRNA and protein levels (Elowitz *et al*, 2002; Paulsson, 2004). In fact, fluctuations in mRNA numbers can significantly exceed what constitutive expression predicts (Poisson statistics) (Blake *et al*, 2003; Raser & O’Shea, 2004), and it was proposed that this originates in short and intermittent activations of the genes called transcriptional bursts. Transcriptional bursting was formalized as a telegraph model (Peccoud & Ycart, 1995), in which a promoter toggles between transcriptionally active (on) and inactive (off) states. The size of the bursts (*b*) represents the average number of transcripts produced during the active period. Recent assays in single cells confirmed transcriptional bursting in many organisms (Golding *et al*, 2005; Chubb *et al*, 2006; Raj *et al*, 2006; Zenklusen *et al*, 2008). Although not all genes are transcribed in bursts (Zenklusen *et al*, 2008), bursting appears predominant in mammals (Suter *et al*, 2011; Dar *et al*, 2012; Bahar Halpern *et al*, 2015). The mechanisms causing bursts in eukaryotes are still elusive but most likely involve the interplay between transcription factors (Larson *et al*, 2013; Senecal *et al*, 2014), chromatin remodelers (Coulon *et al*, 2013; Voss & Hager, 2013), the formation of gene loops and pre-initiation complexes (Blake *et al*, 2003; Zenklusen *et al*, 2008), and transcription initiation and elongation (Jonkers *et al*, 2014; Stasevich *et al*, 2014).

Recent time-lapse imaging to monitor bursting of endogenous mammalian genes (Harper *et al*, 2011; Suter *et al*, 2011) reported peaked silent transcriptional intervals, suggesting a refractory period lasting about 1 h preceding transcription reactivation. Similarly, promoter refractoriness to reactivation was reported in *Neurospora*, indicating a form of molecular memory (Cesbron *et al*, 2015). Refractory periods support a model of promoter progression (Hager *et al*, 2006; Métivier *et al*, 2006) in which sequential metastable changes in the local chromatin template underlie a multi-step progression toward transcription activation (Coulon *et al*, 2013). In first approximation, this promoter progression can be considered as an irreversible cycle (Zhang *et al*, 2012), whose rate-limiting steps need to be estimated, which we address here.

Detailed knowledge on the transcriptional kinetics also allows better understanding of noise in gene expression (Ozbudak *et al*, 2002; Swain *et al*, 2002; Paulsson, 2004; Sanchez & Kondev, 2008), which is relevant notably in the context of RNA-counting experiments in developmental (Little *et al*, 2013; Bothma *et al*, 2014) or cell differentiation systems (Chang *et al*, 2008; Abranches *et al*, 2014; Ochiai *et al*, 2014). Importantly, the structure and kinetics of the promoter cycles will also impact the noise in gene expression since it determines the statistics of the off intervals (Pedraza & Paulsson, 2008). Indeed, in addition to the standard transcriptional parameters (burst size, activation frequency), the number of rate-limiting steps also tunes noise levels (Zhang *et al*, 2012).

Here, we combined temporal single-cell measurements of short-lived and highly sensitive luciferase reporters with mathematical modeling to characterize silent transcriptional intervals. In particular, by modeling promoters as an irreversible cycle, we estimated the number and durations of the rate-limiting steps responsible for refractory periods in mammalian gene reactivation. We found gene-specific structure and kinetics of the promoter cycle. Typically, endogenous promoters showed five sequential inactive steps, while minimal synthetic promoters exhibited only one. Two groups of promoter architecture showed distinct transcriptional kinetics; notably, TATA box promoters had only few inactive steps, independently of their genomic location. Moreover, intrinsic noise in our clones was constrained due to transcriptional bursting, and buffered by additional inactive promoter steps. Finally, we analyzed single-cell RNA-seq in mouse embryonic stem cells (mESCs) to validate genome-wide the prediction that TATA box promoters, owing to their reduced number of promoter steps, showed increased intrinsic noise.

## Results

### Refractory period in gene reactivation modeled by a promoter cycle

**Figure 1:**
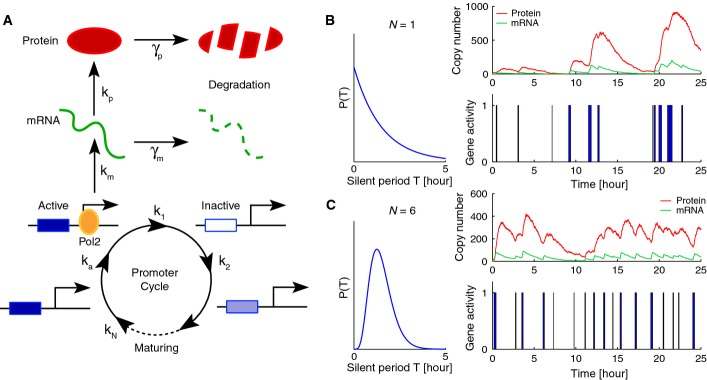
The promoter cycle as a generic stochastic gene expression model to analyze time-lapse imaging data in single mammalian cells The stochastic model describes gene activation, transcription, translation, and degradation of mRNA and proteins. The promoter state follows an irreversible cycle composed of one transcriptionally active state and multiple (*N*) sequential inactive states describing the promoter progression toward activation. Gene-specific rates for the different processes are indicated.

The two-state promoter cycle (telegraph model) predicts exponentially distributed transcriptionally silent periods, yet evidence points toward peaked (non-exponential) durations (Harper *et al*, 2011; Suter *et al*, 2011), which implies out of equilibrium dynamics and irreversibility in the underlying processes (Tu, 2008). A simple yet still sufficiently general model compatible with this constraint is an irreversible *N* + 1-state promoter cycle (Zhang *et al*, 2012), consisting of one transcriptionally active state (on) and *N* sequential inactive states (off), modeling the scenario of promoter progression (Hager *et al*, 2006; Métivier *et al*, 2006). Both the number of states *N* and their durations (not necessarily equal) are not known and will be estimated from data. The resulting stochastic gene expression model (Appendix Supplementary Methods) consists in a two-layered cascade of birth and death processes, describing the production and degradation of mRNAs and proteins (Fig1A). Although this model is a coarse-grained description of gene expression, it accommodates for the observed refractory periods while remaining sufficiently parsimonious to allow inference.

Stochastic simulation of protein numbers, mRNA numbers, and gene activity with *N* = 1 inactive state. Here, the duration of silent intervals is exponentially distributed (left) and the gene expression traces are irregular.

With *N* = 6 states, the duration of silent intervals is now peaked and the expression pattern more regular. Parameters reflect a realistic situation, that is, the average duration of the total silent period *T* is identical in both simulations and set to *T* = 90 min, *τ*_a_ = 8 min and *k*_m_ = 5 mRNA per minutes.

To illustrate the behavior of the model, we compared two realistic simulations differing only in the partitioning of the silent period *T*. A unique step (*N* = 1) yielded exponentially distributed off-times (Fig1B), while partitioning *T* in six subintervals of equal average duration (*N* = 6) followed a peaked (Gamma) distribution. For *N* = 1, we observed large variability in the silent periods. Due to the short active periods, the mRNA and protein time traces were irregular (Fig1B). By contrast, the profiles for *N* = 6 were more regular and the fluctuations in mRNA and protein numbers were reduced (Fig1C), which follows from the more evenly spaced activation events (Pedraza & Paulsson, 2008).

### Identification of optimal promoter cycles

**Figure 2:**
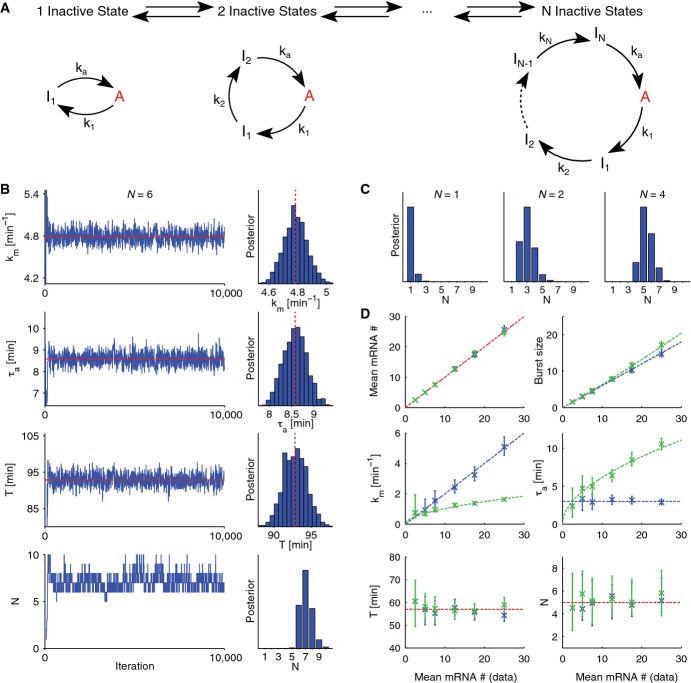
Model selection and parameters estimation based on reversible jump Markov chain Monte Carlo (RJ-MCMC) sampling The number of inactive states in the promoter cycle defines a class of nested models. To sample the different models, we implemented moves (jumps) between models differing by one inactive state.

To characterize the promoter cycles in a set of NIH3T3 cell lines expressing a single allele of a short-lived luciferase reporter driven by different promoters, we extended our computational approach for estimating transcriptional parameters from time-lapse recordings (the transcription rate *k*_m_, the active period *τ*_a_, and the total silent period *T*) (Suter *et al*, 2011; Molina *et al*, 2013) to identify the number *N* and durations *τ*_i_ of transcriptionally inactive states. The translation rate *k*_p_ and the degradation rates of both the protein *γ*_p_ and mRNA *γ*_m_ were measured (Table EV1) and therefore did not need to be inferred. Briefly, we followed a Bayesian approach to estimate the joint posterior probabilities on *N* and the kinetic rates using a reversible jump Markov chain Monte Carlo (RJ-MCMC) algorithm (Green & Hastie, 2009) (Materials and Methods). RJ-MCMC is a model selection method in which more complex models (larger cycles) are naturally penalized, thus avoiding over-fitting. Implementing this scheme requires computing the likelihood of each bioluminescence time trace under a model (specified by *N* and all kinetic rates). For the likelihood, we used calibrated luminescence signals (Suter *et al*, 2011; Molina *et al*, 2013) (Appendix Supplementary Methods) and the transition probabilities between promoter states, mRNA and protein numbers over the 5-min sampling interval, as dictated by the master equation for the promoter cycle. For the RJ-MCMC sampling, we implemented model-crossing jumps by adding or removing inactive states while keeping *T* constant (Fig2A).

Typical MCMC run, here on simulated data (64 individual traces of 48 h each) generated with *N* = 6 inactive steps. Kinetic parameters and number of inactive states *N* are sampled invariably. Left: MCMC traces, note the short burn in period. Right: Histograms reflecting the estimated posterior distributions, these are centered on the mean values (dashed line), and *N* is between 6 and 9 (most probable is *N* = 7).

Posterior distribution for *N*, inferred from synthetic data as in (B) (with 48 cells per condition), with *N* = 1, 2, and 4, respectively, keeping identical mean silent period *T*.

Performance of the inference on individual transcriptional parameters in function of the simulated mean mRNA numbers (48 cells per mean mRNA). The dashed lines represent the expected values. To vary the mean, either only the transcription rate is increased (blue) or both the on-time and the transcription rate (green) are increased. Crosses show the posterior mean and error bars the 5^th^ and 95^th^ percentiles.

To validate the method, we simulated bioluminescence time traces that mimicked our experiments in terms of the number of cells, length of time traces, measurement noise, and sampling rate (Appendix Supplementary Methods), and tested whether *N*, *k*_m_, *τ*_a_, and 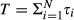 could be recovered. For simplicity, we assumed that the kinetic rates were constant and equal for all cells from the same clone. We estimated posterior distributions of the parameters from four populations sharing identical parameters except *T*, which was partitioned into *N* = 1, 2, 4 and 6 intervals. As exemplified for *N* = 6, we recovered these parameters with good accuracy, albeit with small biases (< 8%) (Fig2B and Table EV2). Similarly, the posterior probability on *N* bracketed the true number, with a tendency to overestimate the most likely value by one (Fig2B and C). To test whether low expression would deteriorate performance, we explored how the mean number of mRNAs and active period *τ*_a_ affect the inference. We generated synthetic populations spanning a realistic range in mRNA expressions (Fig2D) and varied the expression either by changing the transcription rate *k*_m_ or by changing both *k*_m_ and *τ*_a_ (Appendix Supplementary Methods). Remarkably, the recovered parameters were close to the input values even for *τ*_a_ smaller than the 5-min sampling interval and for the lowest expressions (Fig2D and Table EV2). Finally, we tested whether heterogeneous kinetic parameters would affect our estimates. Although inter-cell variability may shorten *τ*_a_ and increase *k*_m_, the burst sizes *b*, *N*, and *T* were not subject to similar biases (Appendix Fig S1). Thus, considering that we used a limited amount of data to mimic the bioluminescence signal and that some parameters describe processes that are filtered at the level of the measured protein expression, we concluded that the inference method performs remarkably well.

### Two groups of gene-specific promoter cycles

We then applied the method to characterize promoter cycles in 16 mouse fibroblasts cell lines (NIH3T3 cells) stably driving a short-lived luciferase reporter from a single allele (Suter *et al*, 2011). These included reporter lines driven by two distinct insertions of the *Bmal1* promoter (B clones); seven clones obtained by lentiviral trapping (gene trap, GT) of endogenous promoters (gene names in Table EV1); and five clones that used the FRT/Flp system to insert into a common location single copies of either the *Dbp* gene (including its promoter) or minimal synthetic promoters combining a TATA box and one (*H1*) or two (*H2*) CCAAT boxes with multiple mutations. Additionally, we generated two more *H1* clones that used new FRT sites in different genomic locations (Appendix Supplementary Methods). Importantly, to minimize transcriptional disturbances during the cell cycle, non-dividing (highly confluent) cells were continuously recorded over approximately 2 days (Chassot *et al*, 2008). We then estimated the transcriptional kinetics from temporal traces in single cells for each clone.

**Figure 3:**
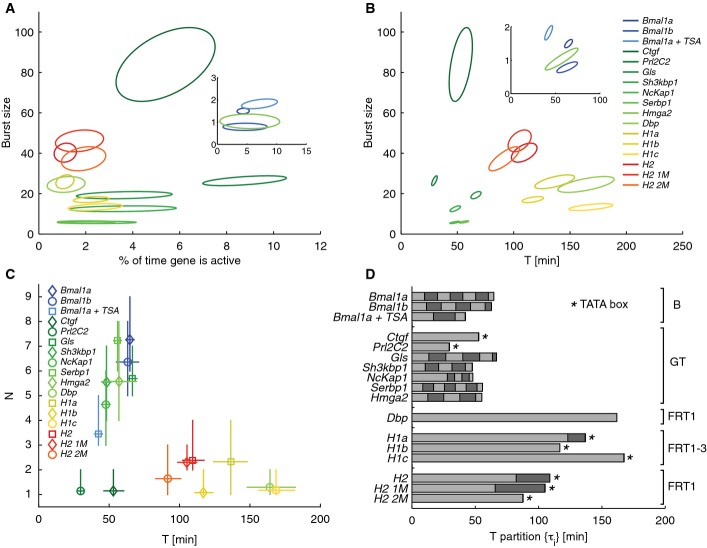
Structure and kinetics of the promoter cycles for the NIH3T3 clones Burst size vs. the fraction of time the gene is active. Each clone is represented by a 95% confidence ellipse from the posterior distribution. All the analyzed clones burst, characterized by small activity fractions. Burst sizes show a large dynamic range across clones (˜80-fold). Inset: Magnification of the lower left corner.

The clones spanned a wide range of burst sizes *b* (from 1 to 80), independent of the fraction of time spent in the active state, which remained under 10% (Fig3A). The infrequent promoter activations clearly indicated that transcription occurs in bursts. Moreover, *b* depended predominantly on the promoter and, to a lesser extent, on the genomic locus, as exemplified by multiple *Bmal1* and *H1* clones. The average duration of the silent period *T* exhibited a smaller dynamic range (from 30 min to 3 h) than the burst sizes, which was the most varying kinetic parameter among the clones (Fig3B). Notably, *b* and *T* appeared largely uncorrelated among the clones. Overall, the extended model yielded kinetic parameters that were largely consistent with previous estimates (Suter *et al*, 2011) (Table EV3). Clearly, the short activation times and large burst sizes implied that transcription in this set of clones is highly discontinuous.

Burst size vs. the total silent period *T*. Elongated confidence ellipses reflect the dependence between those two quantities and the mean mRNA. Although the dynamic range of the silent period (˜6-fold) is smaller than for the burst size, it is also gene specific. The synthetic (warm colors) and endogenous (cold colors) promoters cluster in distinct regions.

Number of inactive states vs. *T*, crosses indicate mean and error bars stand for the 5^th^ and 95^th^ percentiles of the posterior. Endogenous promoters tend to show more inactive steps and shorter cycle times (cluster around *N*˜6 and *T*˜60 min) compared to synthetic promoters (cluster around *N*˜1–2 and *T*˜130 min).

Partitioning of the silent period for the optimal models. The light and dark bars show the mean durations of each sub-step. Partitions in endogenous promoters tend to be more uniform compared to the synthetic promoters. Average inactive times for endogenous promoter are around 10 min, whereas synthetic promoters have average inactive times close to 100 min (˜115 min for the first and ˜25 min for the subsequent intervals).

Examining the structure of the promoter cycles (Fig3C and D), we found that the number of inactive steps *N* differed between the clones (*N *= 1–7). Although it is difficult to gain further insights on the nature of these rate-limiting steps, their timescales of 10 min were more consistent with the dynamics of histone modifications than the interactions of transcription factor with DNA (Discussion). Supporting this, for the *Bmal1* promoter treated with the histone deacetylation inhibitor (TSA), which renders the chromatin more permissive for transcription*, N* reduced from 7 to 3 and *T* reduced from 60 to 40 min (Fig3C and D). The durations of the sub-intervals *τ*_i_ in the endogenous promoters were fairly homogenous, with intervals between 6 and 14 min, whereas synthetic promoters showed one dominating interval. This implied that the silent periods of endogenous promoters should display peaked distributions, whereas the silent periods of synthetic promoters should approximate exponential distributions. To assess the consistency of the inferred promoter cycles with the data, we compared the distributions of silent and active periods from the optimal model with the one obtained using Gibbs sampling (Appendix Figs S2 and S3). Gibbs sampling reconstructs mRNA and gene activity trajectories conditioned on the data in each individual cell using the optimal model as a prior (Appendix Supplementary Methods). It appeared that, for most genes, both the modeled and Gibbs distributions matched closely, confirming the previously observed peaked silent distributions, as well as the aforementioned difference between endogenous and synthetic promoters (Suter *et al*, 2011). Moreover, we did not observe refractory active periods on the scale of the sampling times (Appendix Fig S3).

**Figure 4:**
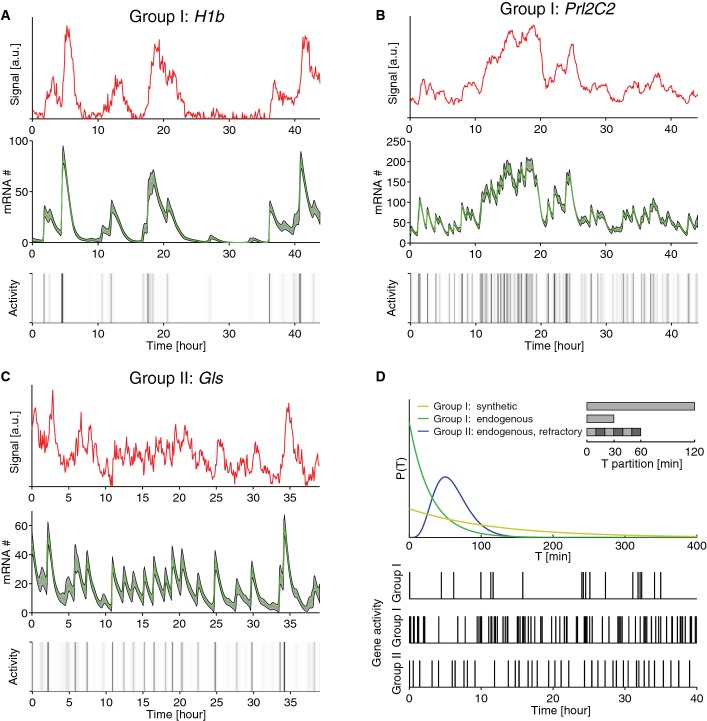
The kinetic structure of the silent intervals reveals different classes of promoters Three classes emerge from the characterization of the promoter cycle: synthetic promoters with a single long silent interval (Group I, synthetic), endogenous promoters with a shorter single interval (Group I, endogenous), and endogenous promoters displaying a refractory period (Group II). A–C Deconvolution of individual cell traces illustrating the three groups of promoter cycles for three promoters (*H1b*, *Prl2C2*, *Gls*). Measured single-cell bioluminescent time traces (red), deconvolved mRNA (green), and gene activity (gray shading indicates probability of the ‘on’ state, black is highest).

Intriguingly, the relationship between *N* and *T* suggested two groups, namely promoter cycles with few steps (Group I: *N* ∼ 1–2) and ones with markedly more steps (Group II: *N* ∼ 6) (Fig3C). In addition, in the first group, all synthetic promoters (six) as well as *Dbp* had long cycles (130 min), while the endogenous promoters (*Ctgf*, *Prl2C2*) had shorter cycles (50 min). Moreover, all promoters with large *N* were endogenous. As shown for representative cells for the *H1* (Group I synthetic), *Prl2C2* (Group I endogenous), and *Gls* promoters (Group II), the distinct kinetics are visible in individual cells, based on the raw signals as well as the mRNA counts and gene activities (Fig4A–C).

D Scheme showing the three groups: Top, distributions of silent intervals. Bottom, Simulations of gene activity patterns for the three groups show qualitative differences.

In summary, the analyzed promoter cycles suggested two distinct groups, simple promoter cycles and complex promoter cycles (Fig4D). Simple promoter cycles (Group I) caused nearly refractory-less and irregular activations, although the irregularity in the endogenous promoters (*Ctgf* and *Prl2C2*) was alleviated by more frequent activations. Complex promoter cycles (Group II) involved several transitions and short silent periods, thus leading to more regular activation patterns constrained by a refractory period.

### Promoter architecture influences the promoter cycles

Since all the synthetic promoters from the original library (*H1a*, *H2*, *H2 1M*, *H2 2M*) were inserted into the same genomic location, the low number of states and long promoter cycle observed might reflect a property of the insertion site, for example, the chromatin state, rather than the promoter architecture. We therefore generated additional clones (*H1b*, *H1c*) by integrating the minimal promoter *H1* at distinct genomic locations. Remarkably, the three *H1* insertions retained very similar promoter cycles (Fig3C and D). While endogenous promoters with similar cycles (Group II) were inherently located in different genomic loci, the two *Bmal1* clones (*Bmal1a*, *Bmal1b*) in two distinct locations also showed very similar cycles (Fig3D), further supporting that the structure of the cycles is primarily a property of the promoters.

Interestingly, the synthetic (Group I) and the two endogenous promoters with small *N* (Group I with the exception of *Dbp*) contained a canonical TATA box element (Dreos *et al*, 2013), which was absent from other endogenous promoters (Group II) with larger *N*. Although the numbers were low, the presence of TATA boxes in promoters with small *N* (in mouse, only < 15% of promoters contain TATA boxes) was non-random (*P*  < 0.01, binomial sampling). Promoter architecture and, in particular, the presence of TATA boxes seemed to influence the promoter cycles. Further evidence that this holds genome-wide is presented below.

### Intrinsic transcriptional noise dominates in non-dividing mammalian cells

**Figure 5:**
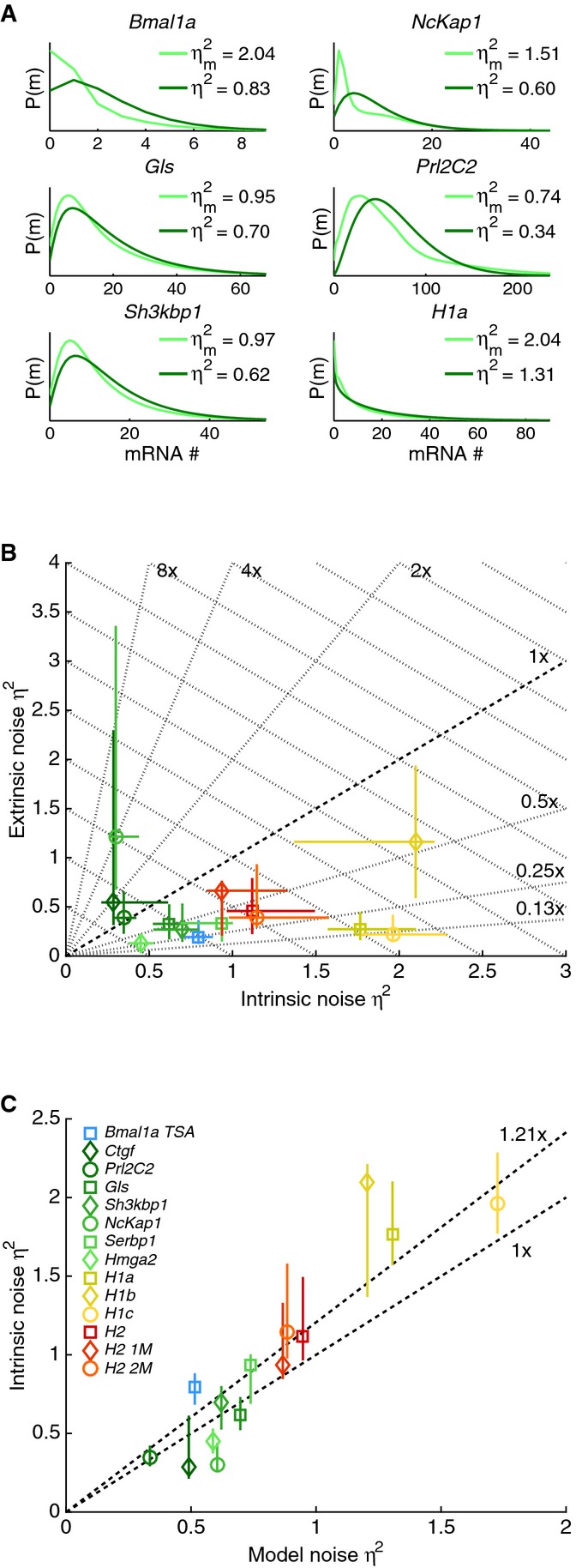
Separating intrinsic and extrinsic noise mRNA distribution (Gibbs, light green) and steady-state distribution of the optimal promoter cycle (dark green). Differences, also reflected in the noise values, originate from extrinsic variability.

We next studied the implications of promoter cycles and transcriptional kinetics on population noise in mRNA numbers, defined as the variance over the mean squared 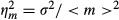 (total noise). Since a fraction of the total noise is expectedly due to extrinsic variability, we split the total noise as 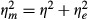. Although this separation can be subtle (Swain *et al*, 2002; Hilfinger & Paulsson, 2011) (Materials and Methods), *η*^2^ (intrinsic noise) arises from gene-specific fluctuations whereas 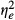 (extrinsic noise) reflects other sources of heterogeneity. To estimate both components for each clone, we used Gibbs sampling to reconstruct the empirical distributions of mRNA numbers in each individual cell (Materials and Methods and Appendix Fig S4). Simulations with heterogeneous cell populations showed that Gibbs sampling accurately recovered the simulated mRNA distributions in each individual cell (and also in the cell population), providing an excellent proxy for 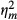 (Appendix Fig S5). In the clones, the empirical population distributions tended to be more dispersed than the fitted model (Fig5A). Indeed for some clones, for example, *NcKap1* or *Ctgf* (Appendix Fig S4B), the model did not capture enrichment at low transcript numbers or longer tails in the empirical distributions. These deviations likely originated from extrinsic noise, such as kinetic parameters differing between cells or over time. Consistent with this interpretation, the circadianly transcribed *Bmal1* promoter showed the largest deviation (Appendix Fig S4B).

Separation of the total noise in intrinsic and extrinsic components. For the majority of clones, intrinsic noise dominates.

The modeled noise corresponds to 83% of the estimated intrinsic noise on average. Data information: In (B, C), the error bars stand for the 5^th^ and 95^th^ percentiles of the estimate (parametric bootstrap).

**Figure 6:**
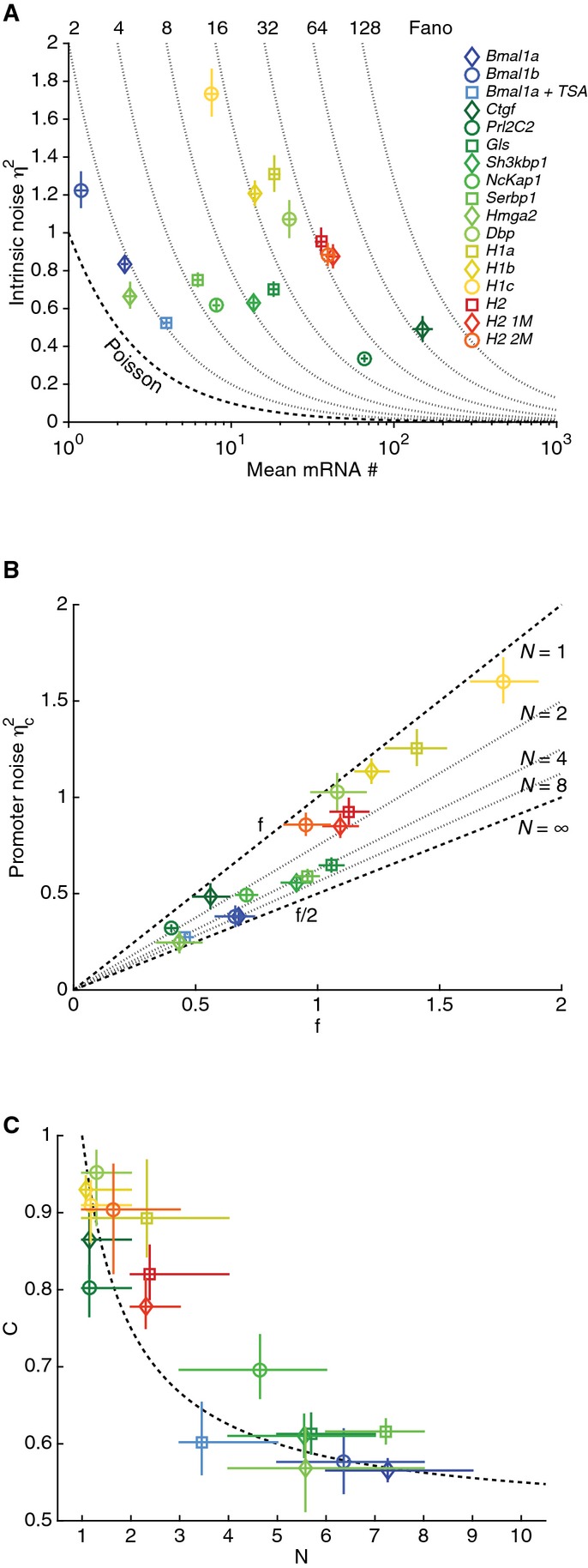
Relationship between mRNA noise and promoter cycles Intrinsic noise *η*^2^ (modeled noise) for the different clones in function of the mean mRNA expression <*m*> (number of copies). The Poisson component 
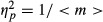
 sets a lower bound on intrinsic noise (lower dashed line). Thus, the promoter noise 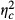 dominates for most genes, as reflected by Fano factors 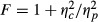 much larger than 2 (thin dotted lines).

The mRNA distributions in individual cells allow splitting of the total variance into the mean variance (proxy for intrinsic variance) plus the variance in the means across cells (extrinsic variance) (Swain *et al*, 2002; Hilfinger & Paulsson, 2011). As verified by simulations (Appendix Fig S5), this split captures *η*^2^ and 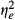 for static cellular heterogeneity (i.e., parameters in each cell remain constant during the recording). Importantly, the recordings were performed in non-dividing cells, removing one important source of temporal heterogeneity (Zopf *et al*, 2013). Since the *Bmal1* and *Dbp* clones are sensitive to circadian oscillations, we restricted our noise analysis to the other clones, except for *Bmal1* treated with TSA, which abolishes circadian oscillations while maintaining transcriptional bursting (Suter *et al*, 2011). In most clones, *η*^2^ exceeded 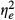 (Fig5B), and *η* (coefficient of variation CV) was on the order of 100% (*η*^2^ between 0.3 and 2.1), independent of expression levels. As shown below, this a direct consequence of transcriptional bursting. In comparison, *η*_e_ was in the range of 70% (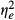 between 0.1 and 1.2) for a majority of clones. Among the few clones dominated by extrinsic noise, *Ctgf* is known to be highly sensitive to stimulations (Molina *et al*, 2013). Importantly, in both the clones and the simulations (Fig5C and Appendix Fig S5D), a high portion of the estimated intrinsic noise (83% on average) was captured by the optimal model (Fig5C), which allows us to study how the noise depends on transcriptional parameters (Fig6).

Promoter noise 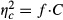 is bounded between *f* = (*τ*_*a*_ + *T*)/*τ*_*m*_ and *f*/2.

The promoter-cycle noise coefficient *C* decreases as the number of inactive states *N* increase and is well approximated by 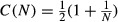, the minimal value for fixed *N* when *τ*_a_ ≪ *T* (bursting). Data information: The error bars stand for the 5^th^ and 95^th^ percentiles of the posterior distribution.

### Transcriptional bursting and promoter cycles constrain noise of mammalian genes

We next investigated how the promoter cycles, in particular the number of steps *N*, affect intrinsic noise *η*^2^ in our clones. Theoretical studies of similar models (Pedraza & Paulsson, 2008; Sanchez & Kondev, 2008; Zhang *et al*, 2012) showed that *η*^2^ separates as 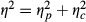, where 
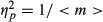
 is the Poisson noise (*p* refers to Poisson), which sets a lower bound on intrinsic noise, and 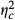 (*c* refers to “cycle”) corresponds to the promoter noise. In the clones, *η*^2^ was larger than 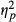 (reflected by the large Fano factors 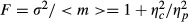) and exhibited only moderate variation (0.34–1.73) over a 100-fold range in mRNA expression (Fig6A). This follows simply from the property that the burst size *b* (from 1 to 80) explains most of the variation in expression (Appendix Fig S6A and B). Indeed, since *b* and *T* are largely independent parameters (Fig3B), the noise in the promoter cycle model is conveniently expressed as 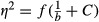 with 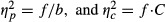. Here, the fraction *f* = (*τ*_*a*_ + *T*)/*τ*_*m*_ is the duration of the promoter cycle, or intervals between bursts, normalized by the lifetime of the transcript *τ*_m_, and sets the scale of the intrinsic noise. The coefficient *C*, originating from promoter fluctuations, approaches zero in a constitutive regime (*τ*_*a*_ ≫ *T*) and is always between 1/2 (large *N*) and 1 (*N* = 1) in a bursting regime (*τ*_*a*_ ≪ *T*). This shows that 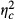 exceeds 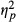 for *b* > 2, which is the case for most clones (Fig3). Thus, fluctuations of the promoter cycle dominated intrinsic noise from a single allele for most promoters.

Since *C* is constrained, *f* explains most of the variance in promoter noise (Fig6B). However, *C* can decrease in two ways, either by changing from a bursting to a constitutive regime (increasing *τ*_a_), or by increasing *N*. Although *C* is a complicated function in general (Appendix Supplementary Methods), it reduces to 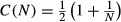 in a bursting regime (*τ*_a_ ≪ T), when the inactive sub-steps have equal durations (*τ*_i_ = *T/N*) and *T* ≪ τ_m_. This limit links with noise 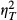 in the cycle duration 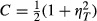 (Pedraza & Paulsson, 2008) because 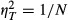 for Gamma distributions. Moreover, this limit coincides with the lowest possible value for a given *N* (optimal noise reduction). It turns out that the different clones are well approximated by *C*(*N*) (Fig6C), with a few clones slightly deviating from the approximation (the ones above the dash line), mainly due to asymmetric partition of the silent period, which is suboptimal in terms of noise reduction (Zhang *et al*, 2012). Overall, the structure of the cycle reduced intrinsic noise in mRNA levels by up to 30% (Appendix Fig S7), which occurred for genes strongly dominated by promoter noise and with large *N*.

Thus, we showed that since mammalian genes are typically transcribed as short and large bursts, the intrinsic mRNA noise was on the order of the normalized promoter cycle duration.

### TATA box promoters exhibit larger intrinsic mRNA noise genome-wide

**Figure 7:**
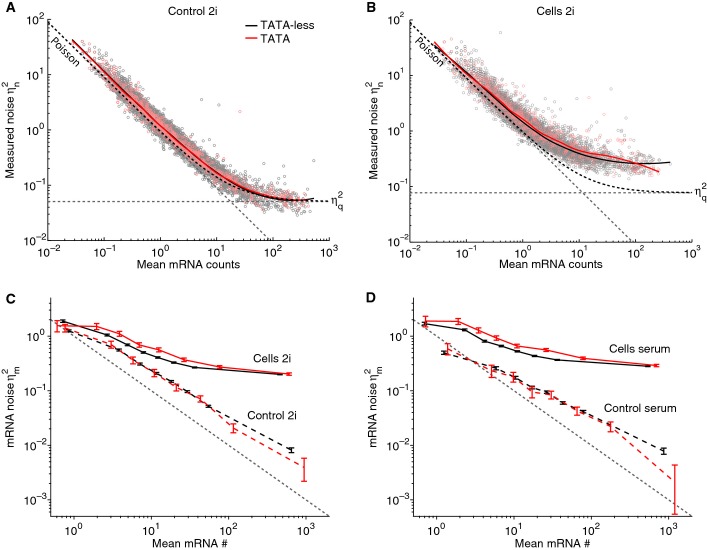
mRNA noise of TATA and TATA-less promoters inferred from single-cell RNA-seq in mouse embryonic stem cells A, B Total mRNA noise vs. average number of mRNA counts for TATA-less (gray dots) and TATA promoters (light red dots) in controls (A) and in cells (B). Gray dashed lines indicate Poisson (slope of −1) and recovery 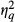 (variability of the recovery rate across cells, horizontal) noises, and the curved black dashed lines indicate the predicted measured noise in the absence of biological noise.

The grouping of promoters according to *N* (Figs3 and 4) predicts that TATA box promoters in general should exhibit increased intrinsic mRNA noise due to a simplified promoter cycle (*N* = 1). In yeast, TATA box promoters are known to exhibit increased noise (Blake *et al*, 2006; Newman *et al*, 2006; Hornung *et al*, 2012), presumably due to distinct nucleosome organization (Raser & O’Shea, 2004; Field *et al*, 2008; Tirosh & Barkai, 2008). Although similar mechanisms should be expected in higher eukaryotes, the role of TATA boxes on mRNA noise in mammals is less studied (Miller-Jensen *et al*, 2013). To test our prediction, we analyzed single-cell RNA-seq data from mouse embryonic stem cells (mESCs) (Grün *et al*, 2014) generated with unique molecular identifiers to reliably count mRNAs. An important parameter required for noise analysis in RNA-seq is the recovery rate *q* (sensitivity), estimated to be around 10%. Indeed, at low counts, non-biological sampling noise (showing Poisson statistics) dominated in both the split controls and the single cells (Fig7A and B), whereas for large counts, the noise plateau was higher in the cells, reflecting additional promoter and extrinsic noise compared to the controls. However, despite the artificially large noise range (two logs) due to low sensitivity, TATA box-containing promoters (Dreos *et al*, 2013) as a group showed subtle but increased noise in the cells, which was most visible in the range of 1–100 measured mRNA counts (Fig7B), and absent in the control (Fig7A). Correcting for the sampling noise showed that TATA promoters, on average, exhibited excess in biological noise of 0.1–0.2 in both 2i and serum conditions, across a significant range in expression (Fig7C and D). For the control, we recovered noise that scaled inversely with the mean, although with a slightly higher magnitude than the expected Poisson noise arising from the re-splitting of mRNAs from pooled cells. A parsimonious explanation is that the higher intrinsic noise in the TATA promoters reflects the promoter switching kinetics. Of note, comparing genes on the X (one allele) with genes on autosomal chromosomes (two alleles), where the effective promoter noise from the two alleles is predicted to be lower, showed a similar difference (Appendix Fig S8A and B). Quantitatively, the promoter cycle model predicts that in bulk (taking an average *f*), the difference in promoter noise between TATA (*N* = 1, *C* = 1) and TATA-less promoters (*N* large, *C* ∼ 1/2) amounts to *f*/4 (due to the two alleles). The same quantitative difference is predicted for genes on the autosomal vs. X chromosomes (assuming *C* ∼ 1/2 for endogenous genes). Gene-specific values of *f* are not known but estimated between 0.01 and 0.5, based on promoter cycle times in the range of 1–2 h (Fig3) and half-lives in the range of 1–20 h (Sharova *et al*, 2009), which is at least consistent with the observed difference of 0.1–0.2. Notably, transcript half-lives are not significantly different for TATA and TATA-less promoters (Sharova *et al*, 2009) (not shown). The common noise plateau for TATA and TATA-less promoters (Fig7C and D), which is also observed for X vs. autosomal genes (Appendix Fig S8A and B), suggested that the promoter noise is negligible at high expression. A plausible explanation could be that *C* goes to zero at high expression due to constitutive expression (Sanchez *et al*, 2013). This would imply that extrinsic noise for highly expressed genes in the mESCs averages about 0.25 (CV = 50%) in the 2i conditions and 0.35 (CV = 60%) in serum, consistent with the higher phenotypic heterogeneity in serum. Incidentally, these values were of the same order as the extrinsic noise estimated for our 3T3 clones (Fig5) using a radically different approach.

C, D Sampling corrected noise in function of mRNA copy number for two medium condition (two inhibitors 2i, C) and serum (D) for TATA-less promoters (black) and TATA promoters (red). Error bars correspond to the 5^th^ and 95^th^ percentiles of the posterior distribution. The gray dash line represents the Poisson noise; residual Poisson noise in the controls reflects splitting variability.

## Discussion

How mammalian transcription in single cells performs complex regulatory tasks reliably given the nano-scale machineries involved is tantalizing. Recently, real-time monitoring of transcriptional fluctuations provided new dynamical insights into the underlying molecular processes, while also revealing physical limits on the expected precision. From time-lapse transcriptional recordings of endogenous and synthetic mouse promoters, we identified minimal models of promoter cycles, estimated the durations of rate-limiting steps underlying refractory periods, and studied the consequences on expression noise.

### Modeling promoter cycles

The recent possibility to quantitatively model transcriptional recordings in single mammalian cells revealed that transcriptional kinetics bears the signature of refractory promoter states (Harper *et al*, 2011; Suter *et al*, 2011; Molina *et al*, 2013). Here, we developed a model selection approach on a class of promoter cycles to further characterize the number and durations of rate-limiting steps underlying the refractory state. As these properties are essentially reflected in the distributions of silent transcriptional periods, time-lapse approaches (destabilized reporters, MS2-GFP) offer significant advantages over static methods (FACS, RNA-FISH, RNA-seq) (Stinchcombe *et al*, 2012), since both the size and temporal correlations of the transcriptional fluctuations are available. Indeed, explicitly modeling bioluminescence time traces enabled us to resolve the kinetic structure of the cycle on relatively short timescales compared to the sampling time (Fig2D). While the bioluminescence approach offers several advantages (e.g. sensitivity and long term recordings), one limitation is that promoters kinetics are inferred from protein time series, which entails additional assumptions compared to more direct methods such as MS2-GFP (Yunger *et al*, 2010; Larson *et al*, 2013). Namely, to perform the inference, we assumed minimal models of gene expression, in which the promoter dynamics is described by transitions between discrete, transcriptionally active and inactive, promoter states. In addition, the promoter dynamics follow a promoter progression (Larson, 2011; Coulon *et al*, 2013). In this scenario, interactions of transcriptional regulators and various cofactors with DNA induce a temporally ordered sequence of modifications in the chromatin template, eventually leading to a transcriptionally active state, whose lifetime is finite. The resulting promoter reaction scheme consistent with sequential and time-ordered transitions takes the form of an irreversible cycle (Zhang *et al*, 2012). Moreover, downstream of transcription, we did not explicitly model fast processes, such as mRNA and protein maturation, nuclear and cytoplasmic transport. Such a coarse-grained description remains valid as long as the omitted processes are rapid compared to the explicitly modeled reactions. Although identifying which processes may be rate-limiting is *a priori* challenging (Pedraza & Paulsson, 2008), confronting these models with real data using the developed framework provides a constructive approach to eventually refine the models.

### Nature of the rate-limiting steps

Since the timescales of the rate-limiting steps involved in promoter cycles were on the order of ten minutes, these do not likely reflect transcription factor–DNA interactions. Indeed, such dynamics in mammals is often faster, with mean search and residence times in the range of a few seconds to one minute (Mazza *et al*, 2012; Gebhardt *et al*, 2013; Izeddin *et al*, 2014). On the other hand, histones can carry longer lasting metastable modifications, which could provide a basis for a slow multistep process (Coulon *et al*, 2013; Voss & Hager, 2013). Consistent with the implication of histone modifications, *Bmal1* treated with TSA exhibited a reduced number of steps and shorter silent period. The distinct promoter dynamics shown by the two groups of clones might reflect promoter-specific chromatin properties, such as nucleosome organization, and related changes in chromatin conformation required for initiation (Sanchez *et al*, 2013). In mammals, TATA box promoters have precisely defined TSSs usually covered by a nucleosome (Lenhard *et al*, 2012). Competition between the nucleosome and the TATA-binding protein could function as a simple switch (Hapala & Trifonov, 2013; Hieb *et al*, 2014), as shown in yeast (Field *et al*, 2008; Tirosh & Barkai, 2008), and might explain the single rate-limiting step. In general, nucleosomes at active yeast and mammalian promoters undergo frequent turnover (Raser & O’Shea, 2004; Dion *et al*, 2007; Huang *et al*, 2013; Kraushaar *et al*, 2013) with timescales of 25 min, which is compatible with the inferred steps. Refractory periods in mammalian gene reactivation imply non-equilibrium dynamics (Tu, 2008) and energy consumption, consistent with sequentially regulated and ATP-dependent chromatin transitions (Coulon *et al*, 2013). In principle, this energy consumption could be estimated directly from the kinetic structure of promoter cycles (Schnakenberg, 1976), provided that the cycle incorporates both forward and backward reactions. This might, however, be difficult in practice using our approach due to additional parameters in the models.

### mRNA and protein noise

A central question in stochastic gene expression is to understand how the underlying molecular events influence the coefficient of variation (or noise), either across cells or over time (Thattai & van Oudenaarden, 2001; Paulsson, 2004). In particular, how noise depends on the mean mRNA and protein levels has attracted significant attention (reviewed in Sanchez *et al*, 2013). In general, noise decreases with increasing expression, but how exactly it scales with expression, and over which range, is to a large extent encoded in the way that expression levels are changed. This can occur at many levels, for example by changing either the burst frequencies or burst sizes. Interestingly, it appears that different organisms use different strategies. Namely, in yeast, burst sizes seem fairly constrained and mostly independent of the mean expression (Hornung *et al*, 2012; Carey *et al*, 2013; Sanchez & Golding, 2013), which explains why expression noise is inversely proportional to the mean expression over most of the range (Bar-Even *et al*, 2006; Newman *et al*, 2006). By contrast, in mammals, it was shown for the HIV promoter integrated at different genomic loci in human cells that increased expression reflects increased burst frequency up to intermediate levels, followed by an increase in burst sizes at high levels (Dar *et al*, 2012). Using our reporters, we showed that burst sizes in mouse are highly correlated with expression (Suter *et al*, 2011; Dar *et al*, 2012), which we consolidate in this study (Appendix Fig S6A). This implies that noise in mammalian genes does not scale inversely with expression over the entire range, but in fact flattens out starting at relatively low expression, namely above about 10 mRNA copies, as found both from the time-lapse analysis (Fig6A) and from RNA-seq (Fig7C and D). While such a plateau could also reflect extrinsic noise (Bar-Even *et al*, 2006), we here argued that this residual noise (CVs between 0.5 and 1.3) originates from promoter fluctuations. Moreover, it depends on the kinetics of the promoter cycle and lifetime of transcripts, but not on the transcription rate, and is reduced by two-fold at most when the number of promoter steps is high (Fig6C). Interestingly, we found that mammalian TATA box genes exhibited a single rate-limiting step in promoter reactivation, and thus higher promoter noise than TATA-less genes by virtue of the promoter coefficient *C*. A similar difference in noise between promoter architectures has been extensively studied in yeast (Newman *et al*, 2006; Hornung *et al*, 2012; Sharon *et al*, 2014), although there, the increased noise in TATA-containing genes has been attributed to increased burst size rather than via the coefficient *C* reflecting promoter switching dynamics.

Both the mRNA and protein in our single allele reporters were destabilized; however, we can estimate how typical endogenous half-lives (Sharova *et al*, 2009; Schwanhäusser *et al*, 2011) would affect noise. Although the longer endogenous half-lives lead to higher expression and thereby buffer mRNA noise by virtue of the factor *f*, the fraction of intrinsic noise from the promoter (equivalent to the Fano factor minus one) would be nearly insensitive. Moreover, the reduction of noise from a larger number of rate-limiting steps in the promoter cycle would be close to optimal for long-lived transcripts (Appendix Supplementary Methods). Also, for endogenous genes on autosomal chromosomes, the presence of two uncorrelated alleles (Hocine *et al*, 2013; Deng *et al*, 2014) would reduce the promoter noise by another factor of two (Raj *et al*, 2006). Finally, since mRNA noise is propagated almost linearly to the proteins for realistic parameters (Appendix Fig S9A) (Pedraza & Paulsson, 2008), the noise reduction by the promoter cycles transposes to the protein level (Appendix Fig S9B).

### Signatures of promoter fluctuations in single-cell RNA-seq

Our time-lapse analysis predicted that TATA box promoters would exhibit higher promoter noise due to their low number of promoter steps. To test this genome-wide, we analyzed single-cell RNA-seq in mESCs. Surprisingly, despite a number of confounding factors such as technical variability in the library preparations, sampling bias, and extrinsic noise, which could have masked the promoter effects, we found that RNA-seq experiments (Grün *et al*, 2014) revealed signatures that were consistent with intrinsic biological noise. In particular, the presence of TATA box promoters or gene dosage (Halpern *et al*, 2015) affected promoter noise, with effect sizes that were in the expected range (Fig7). This may explain how tissue-specific mammalian genes, which are often linked with TATA boxes (Lenhard *et al*, 2012), showed increased mRNA noise (Padovan-Merhar *et al*, 2015). While the interpretation in terms of intrinsic noise is the most parsimonious, we cannot entirely exclude that differential susceptibility of TATA box genes to extrinsic fluctuations may also contribute. While increased noisiness in TATA box promoter has been widely studied in synthetic and endogenous genes in yeast (Sanchez *et al*, 2013), our results generalize this to mammalian genes, identifies its origin in the promoter noise, and provides a simple explanation in the structure of the promoter cycle.

### Conclusion

We combined time-lapse transcriptional measurement in single mammalian genes with mathematical modeling to estimate the durations of rate-limiting promoter steps underlying promoter refractoriness. This analysis further indicated that the transcriptional and noise properties of the promoters are encoded primarily in *cis*, with promoter architecture playing a key role in shaping gene expression noise.

## Materials and Methods

### Single-cell time-lapse data

Single-cell time-lapse recordings of single-copy destabilized luciferase reporters in NIH3T3 fibroblasts were taken from a previous set of stable clones (Suter *et al*, 2011), complemented by two newly generated *H1* clones inserted in new FRT sites (Table EV1 shows the full list of analyzed promoters, measured translation, and degradation rates). For the new H1 clone generation and microscopy settings, see Appendix Supplementary Methods. All single-cell time traces analyzed in this study are provided in Dataset EV1.

### Likelihood calculation

To perform inference, we computed the exact likelihood that bioluminescence time traces were generated by a stochastic gene expression model describing the promoter progression toward activation, followed by synthesis and degradation of mRNAs and proteins. The promoter cycle constituted of *N* sequential inactive states and a unique active state in which mRNA transcription may occur. The likelihood of a single luminescent time trace of length 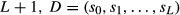, given *N* inactive states and kinetic parameters *θ*_N_, is 

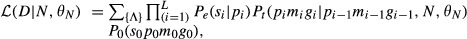
where *P*_e_(*s*¦*p*) stands for the probability to measure *s* gray levels given *p* protein copies and follows from our previous microscope calibration (Suter *et al*, 2011). The transition probabilities *P*_t_(*pmg*¦*p*’*m*’*g*’,*N*,θ_N_) are derived from the master equation, where the discrete state *pmg* stands for the protein (*p*), mRNA (*m*) copy numbers, and the state of the promoter *g*. *P*_0_(*s*_0_*p*_0_*m*_0_*g*_0_) corresponds to the stationary distribution, and the sum runs over all possible state trajectories Λ = {*pmg*}.

### Model selection and parameters estimation

Using the likelihood, the optimal model was inferred from the joint posterior distribution *P*(*N*,θ_N_¦*D*), 

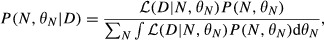
where *P*(*N*,θ_*N*_) is the prior distribution and 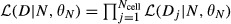 the likelihood for an ensemble of cells. To keep the number of parameters manageable, we used global kinetic parameters for all cells in one clone. We sampled *P*(*N*,*θ*_*N*_¦*D*) using a reversible jump Markov chain Monte Carlo (RJ-MCMC) algorithm (Green & Hastie, 2009) that extends standard Metropolis–Hastings sampling to parameter spaces of varying dimension. To sample the nested class of promoter cycles, we designed trans-model moves by either removing the shortest step or randomly adding a short step. At each iteration, new parameters are proposed according to trans-model or within-model moves, and the chain is updated following an acceptance–rejection scheme characteristic of the MCMC approach, which guaranteed detailed balance. The optimal model is selected from the marginal distribution *P*(*N¦D*), and the kinetic parameters are estimated from the marginal *P*(*θ¦D*).

### Deconvolution of time traces

From the optimal model and kinetic parameters, the bioluminescent time traces are deconvolved using Gibbs sampling (Gelfand & Smith, 1990) to reconstruct the mRNAs and gene activity time traces. This approach reconstructs probable state trajectories Λ given the data *D*, using the optimal model as a prior *P*(Λ¦*N*, θ_*N*_). Thus, we sampled from *P*(Λ¦*D, N*, θ_*N*_). given by 

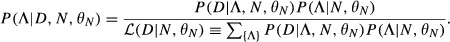


The empirical distribution of silent periods and the mRNA steady-state distribution can be estimated directly from *P*(Λ¦*D,N*, θ_*N*_), while the modeled distributions can be analytically calculated from the promoter cycle model *P*(Λ¦*N*,θ_*N*_). All details on the modeling are given in Appendix Supplementary Methods.

### Transcriptional noise

The total mRNA noise is defined as 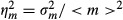. In a static cellular environment (static heterogeneity), the total variance can be split as follows: 

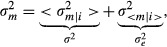
where 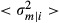 is the average variance over the cell population, and 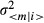 is the variance of the mean expression <*m*¦*i*> in cell *i* across the population. 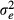 defines the extrinsic variance, while the remaining *σ*^2^ approximates the intrinsic variance of an idealized cell with parameters close to the population mean (Appendix Fig S5, Appendix Supplementary Methods). Both terms can be readily estimated by Gibbs sampling, allowing separation of transcriptional noise 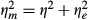.

The intrinsic noise can be further separated as 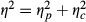, where 
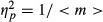
 corresponds to the Poisson fluctuations from the stochastic production and degradation of mRNAs, and 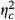 stands for the fluctuations in the promoter cycle 

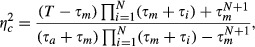
with *N* the number of inactive states, 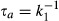 the average active time, 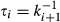 the average time spent in the *i*^th^ inactive state (with *k*_*N*+1_ ≡ *k*_*a*_), 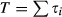 the total silent period, and 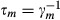 the mRNA lifetime.

### Noise analysis of single-cell RNA-seq

Considering that the recovery rate *q* varies from cell to cell, the expected population noise 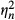 on the counted mRNAs (*n*) relates to the total biological noise 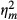 as 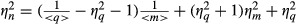, with <*q*> and 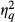 the mean and noise (CV squared) on *q*, respectively. For low *q*, the first term (non-biological sampling noise) typically dominates over the biological noise (Fig7A and B). Expressed in function of the number of counts and taking 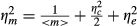 (the 2 arise from the two alleles, which does not reduce extrinsic noise), the sampling and Poisson terms combine, and we find 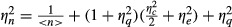 for the single cells, and 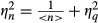 for the splitting controls. Knowing <*q*> and 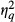 (Grün *et al*, 2014), these expression allow us to extract the expected biological noise (Fig7C and D, Appendix Supplementary Methods). MATLAB scripts to reproduce Fig7 are provided as Code EV1.

## Supporting Information

**Appendix**

**Table EV1**

**Table EV2**

**Table EV3**

**Dataset EV1**

**Code EV1**

**Review Process File**
