# Supplementary material for: Structure of silent transcription intervals and noise characteristics of mammalian genes
Source: Mol Syst Biol. 2015 Jul 27;11(7):823. doi: 10.15252/msb.20156257 (PMC4547851; doi:10.15252/msb.20156257)
Supplement: Supplementary file 1 [file msb0011-0823-sd1.pdf]

Structure of silent transcription intervals and noise characteristics of mammalian genes
Appendix

Zoller Benjamin, Damien Nicolas, Nacho Molina, Felix Naef
Laboratory of Computational Systems Biology,
Institute of Bioengineering, School of Life Sciences,
Ecole Polytechnique Fédérale de Lausanne (EPFL), CH-1015

June 30, 2015

Contents

1 Supplementary Figures **3**

2 Real-time measurements of transcription in mammals **10**
2.1 Suter et al. data 10
2.2 New data 10

3 Stochastic modeling of mammalian gene expression **12**
3.1 Extension of the Telegraph model: the promoter cycle 12
3.2 Likelihood calculations 12
3.2.1 Transition probabilities 13
3.2.2 Efficient likelihood computation 15

4 Model selection and parameters estimation **17**
4.1 Parametrization of the kinetic model 17
4.1.1 Symmetry of the promoter cycle 17
4.1.2 Parameterization of the cycle 18
4.2 Prior distributions 19
4.2.1 Priors on kinetic parameters 19
4.2.2 Prior on the number of inactive steps 20
4.3 Reversible Jump MCMC 21
4.3.1 Move sets & proposal distributions 22
4.3.2 Sampling procedure & error estimates 24

5 Performance **25**
5.1 Inferring the silent partition . . . 25
5.2 Validation with synthetic data . . . 26
5.3 Parameters of each clone & optimal model . . . 28

6 Silent period, active period & steady-state distribution **29**
6.1 Gibbs sampling . . . 29
6.2 Modeled distribution of silent & active periods . . . 30
6.3 Modeled steady-state distribution of mRNA . . . 31
6.4 Inferred vs modeled distributions . . . 31

7 Biological noise **33**
7.1 Transcriptional noise of the promoter cycle . . . 33
7.2 Protein noise . . . 37
7.3 Separation of extrinsic & intrinsic noise . . . 38

8 Single-cell RNA-seq **41**
8.1 Modeling single-cell RNA-seq . . . 41
8.2 Inference of biological noise genome-wide . . . 42

9 Supplementary References **45**

1 Supplementary Figures

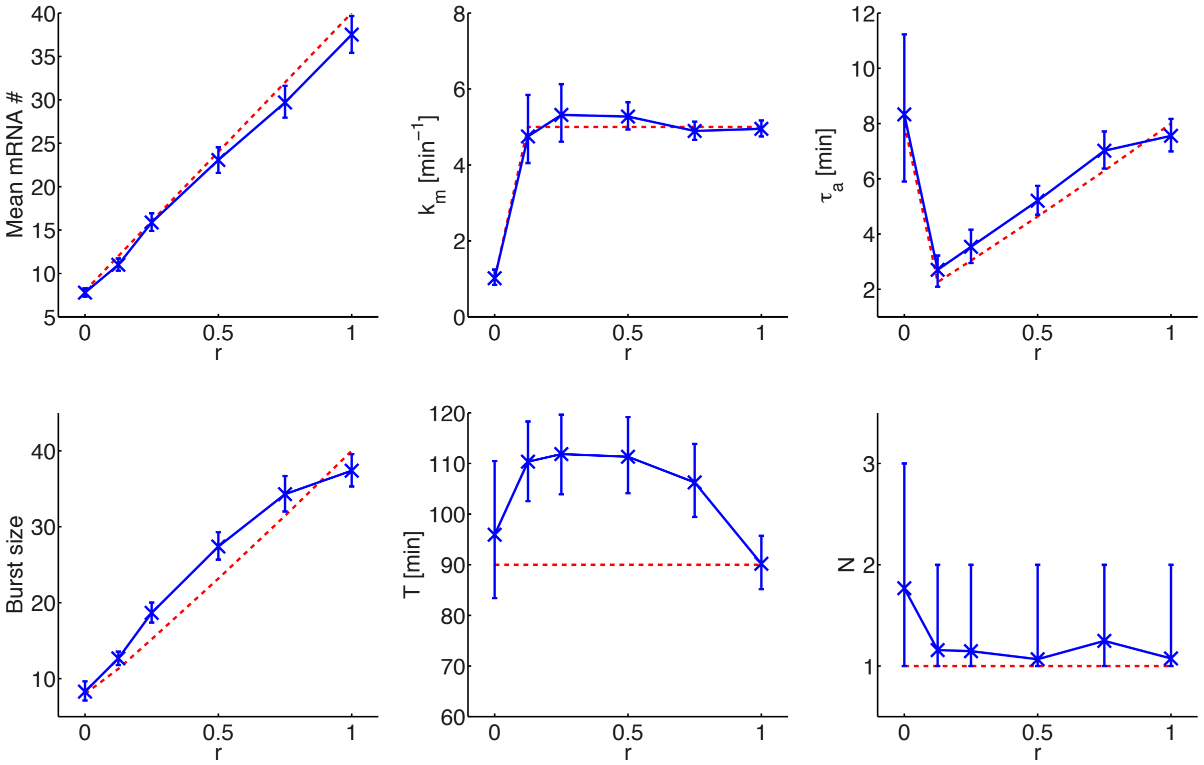

Figure S1: Parameter estimation in heterogenous synthetic cell populations. We generated populations of 48 cells, resulting from the mixing of two data set sharing identical kinetic parameters except the transcription rate k_m, which was set to $k_m^{(1)} = 1$ for the first set and to $k_m^{(2)} = 5$ for the second one. The control parameter r measures the fraction of cells from the second set in the mixed population. The blue crosses stand for the mean parameters in function of r and the error bars for the 5th and 95th percentiles of the posterior distributions. The red dashed lines corresponds to the predicted behavior of the inferred parameters based on the hypothesis that the mean mRNA level $\langle m \rangle$ of the mixed population is correctly inferred and that the estimated transcription rate is actually the maximum among the underlying populations.

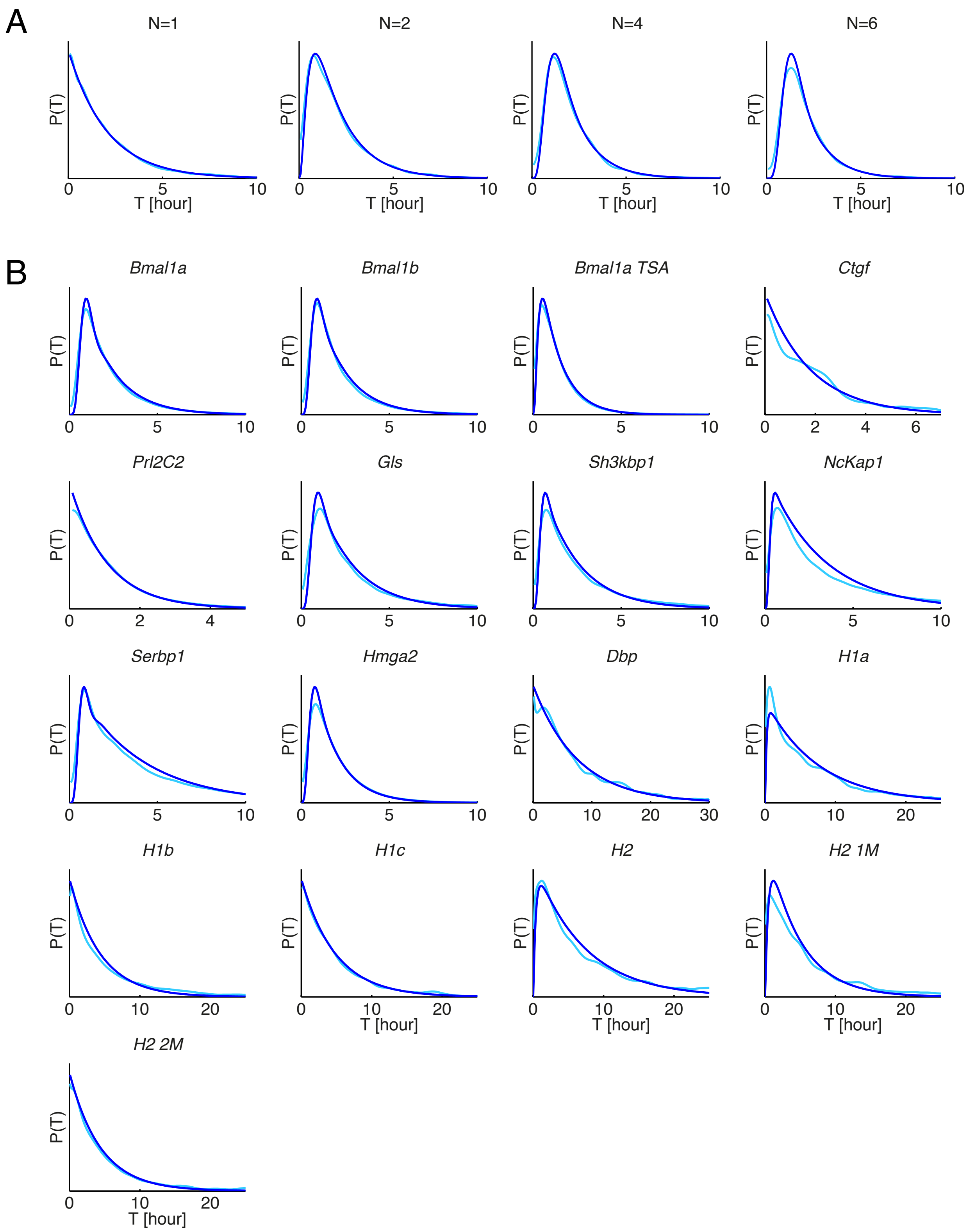

Figure S2: Gibbs sampling reconstruction of the silent period for synthetic data and the full set of clones. Silent period distributions sampled with Gibbs are displayed in light blue, while the theoretical distributions predicted by the optimal model are shown in dark blue. (A) Silent period distributions obtained for synthetic data. We generated 4 data sets of 64 cells, sharing similar kinetic parameters except that the silent period was partitioned in $N = 1, 2, 4$ and 6 silent intervals, respectively. (B) Silent period distributions for each clone.

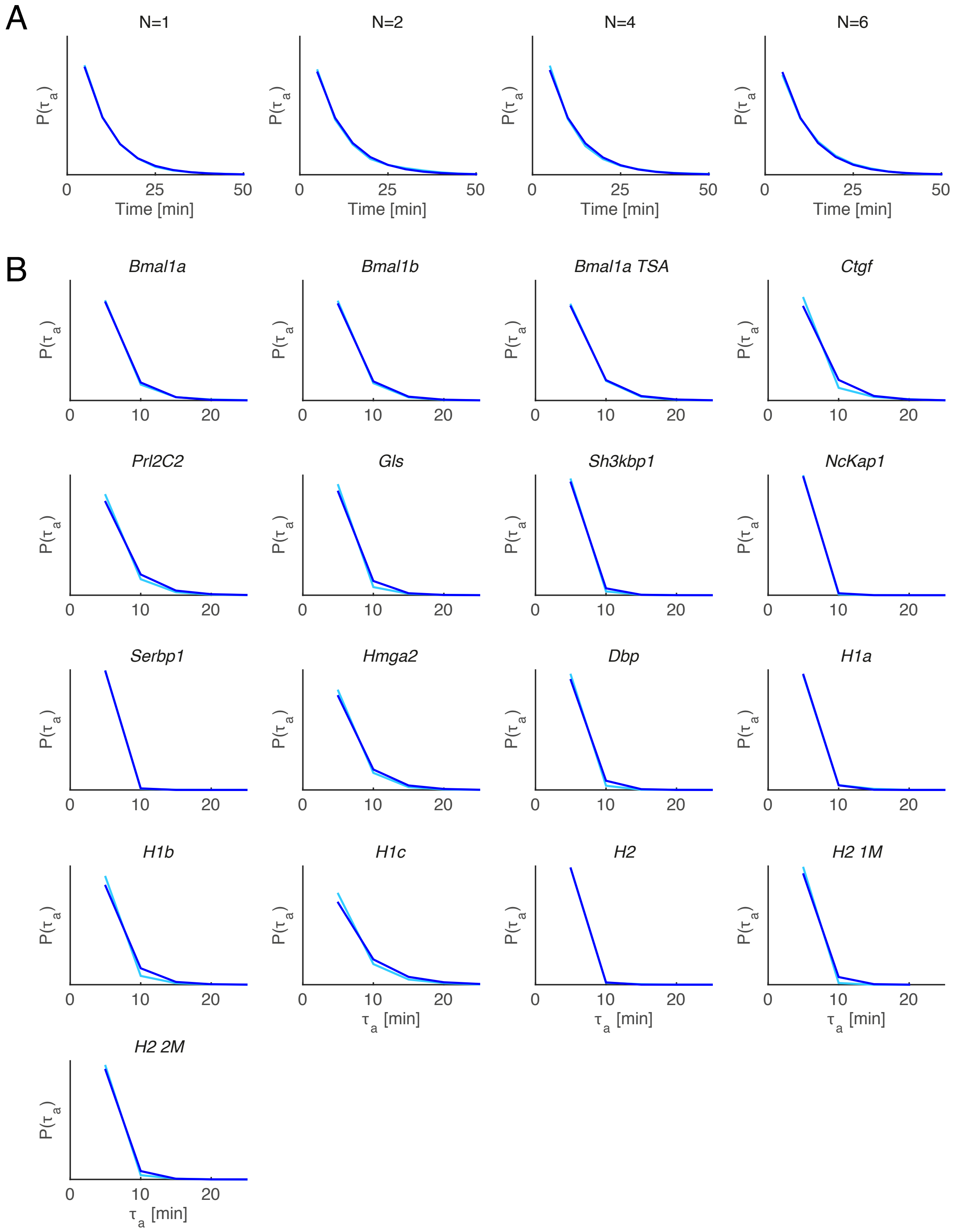

Figure S3: Gibbs sampling reconstruction of the active period for synthetic data and the full set of clones. Active period distributions sampled with Gibbs are displayed in light blue, while the theoretical distributions predicted by the optimal model are shown in dark blue. (A) Active period distributions obtained for synthetic data. We generated 4 data sets of 64 cells, sharing similar kinetic parameters except that the silent period was partitioned in $N = 1, 2, 4$ and 6 silent intervals, respectively. (B) Active period distributions for each clone.

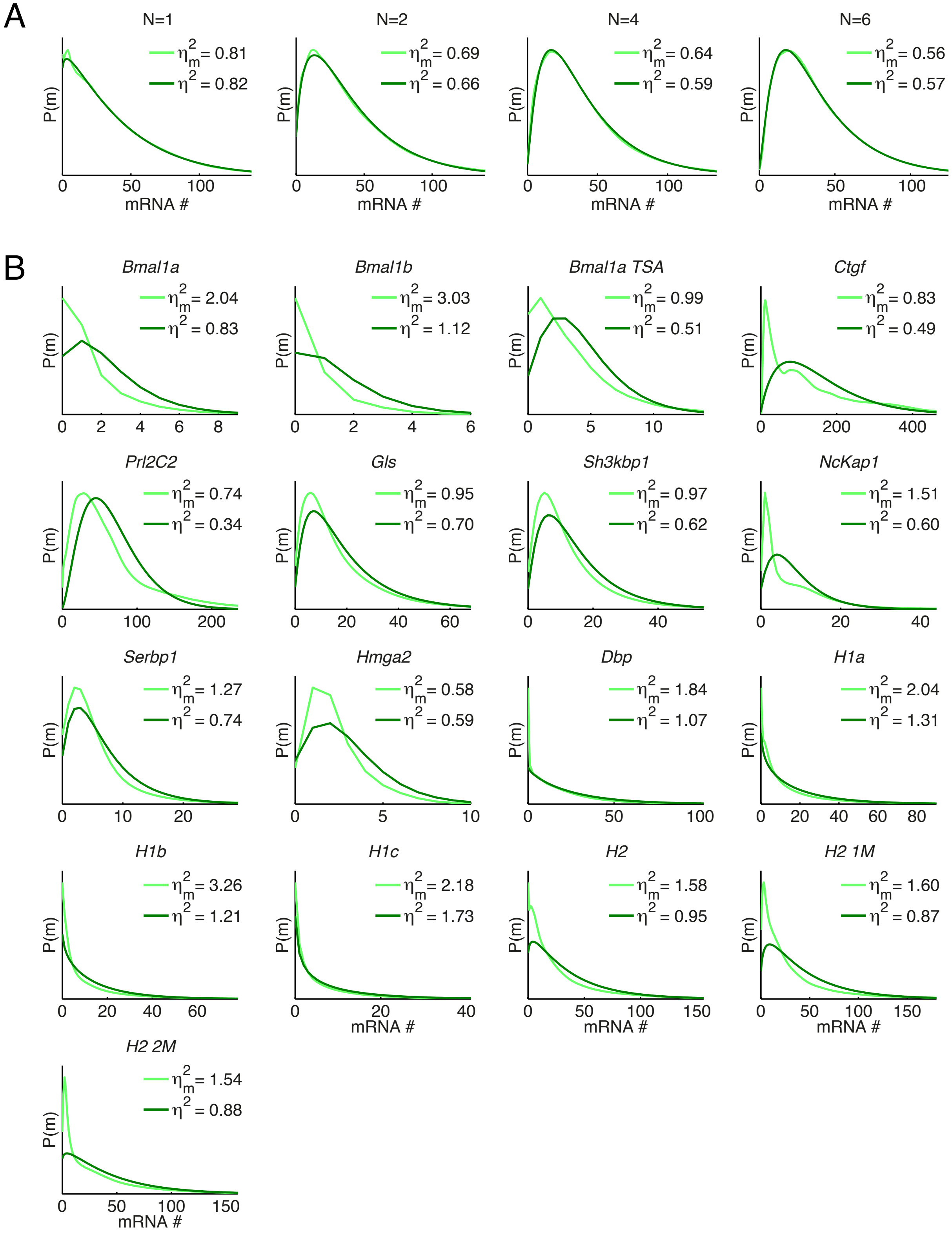

Figure S4: Gibbs sampling reconstruction of the mRNA steady state distributions for synthetic data and the full set of clones. Steady-state distributions sampled with Gibbs are displayed in light green, while the theoretical distributions predicted by the optimal model are shown in dark green. (A) Steady-state distributions obtained for synthetic data and noise values. We generated 4 data sets of 64 cells, sharing similar kinetic parameters except that the silent period was partitioned in $N = 1, 2, 4$ and 6 silent intervals, respectively. (B) Steady-state distributions for each clone and noise values.

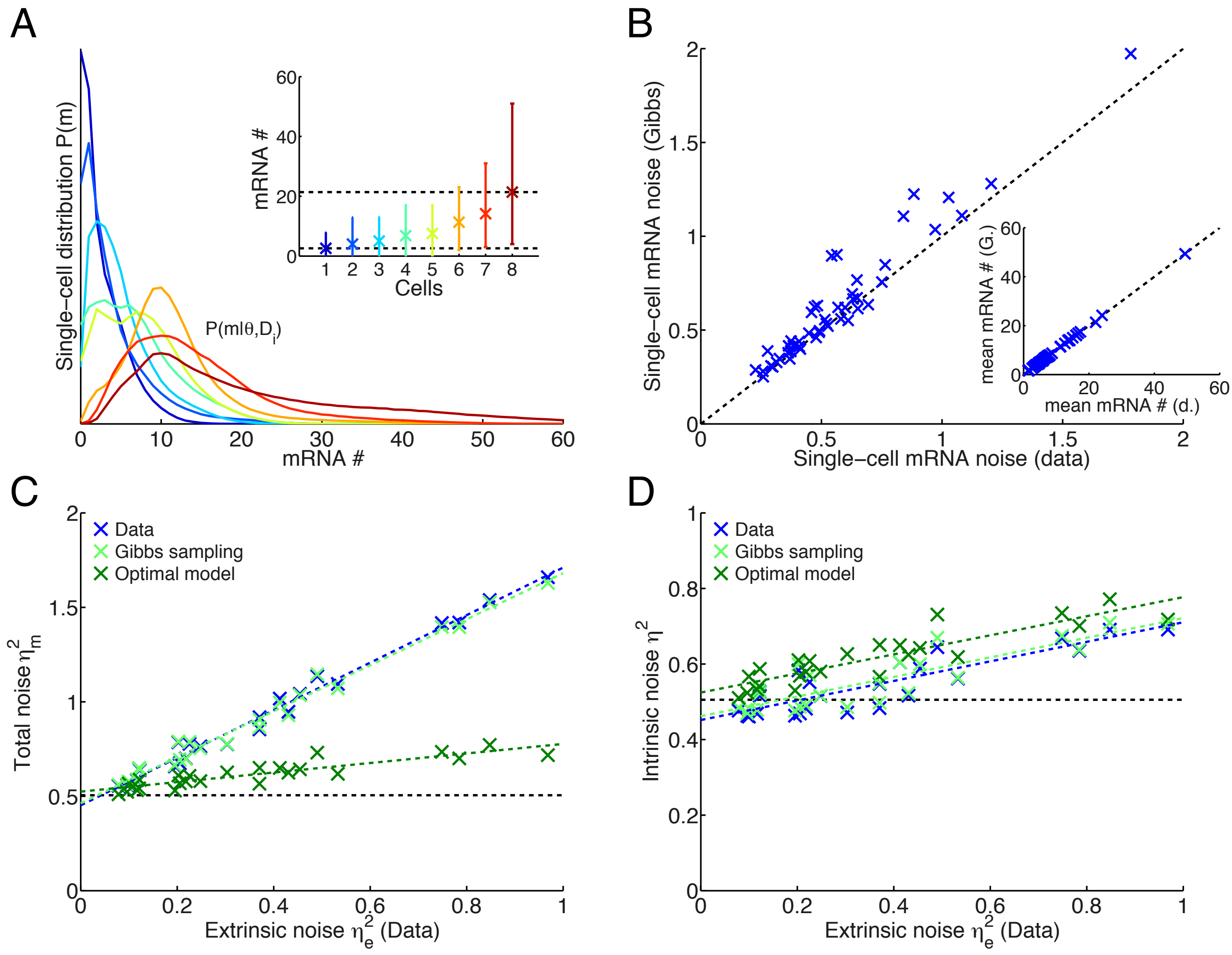

Figure S5: Separation of extrinsic and intrinsic transcriptional noise in synthetic cell populations. (A) Single-cell mRNA distribution obtained by Gibbs sampling for eight representative simulated cells from a population with heterogenous kinetic parameters. Gibbs sampling enables reconstruction of single-cell mRNA distributions $P(m|\theta, D_i)$ conditioned on the data using the model as a prior. Separation of the noise is performed by estimating the mean and the variance of each single-cell distribution (inset: mean, 5th and 95th percentiles of each single-cell distribution). (B) Comparison between single-cell noise estimated with Gibbs sampling and the true single-cell noise in the data for heterogenous simulated population of 48 cells. Inset, comparison between single-cell mean mRNA number estimated with Gibbs sampling and the true value. Overall Gibbs sampling recovers accurately the true single-cell mRNA mean and variance using the optimal model (with global parameters) as a prior. (C-D) Gibbs sampling estimates (light green crosses) and true values (blue crosses) for the population total (C) and intrinsic (D) transcriptional noise in function of the amount of extrinsic noise in the 24 synthetic populations of 48 cells. Noise values predicted by the optimal model for the different population are shown as dark green crosses. The black dash line stand for the expected noise in absence of extrinsic variability (constant kinetic parameters) and the colored dash lines for the linear regression. Gibbs sampling gives an excellent estimation of the total and intrinsic noise in the populations whereas the optimal model gives a close estimation of the true intrinsic noise independently of the amount of extrinsic noise in the data.

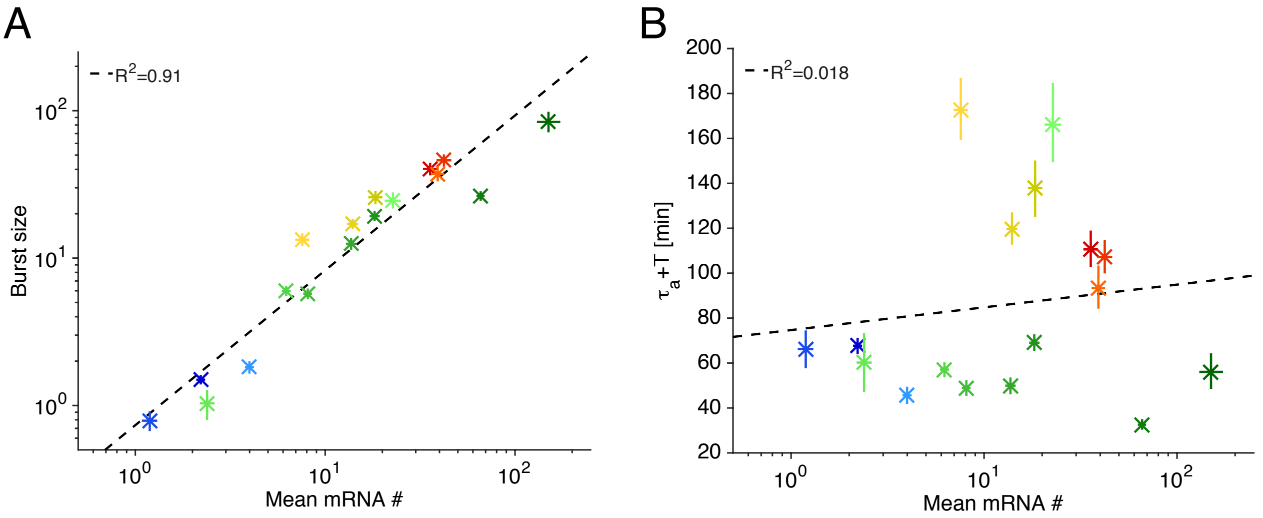

Figure S6: Kinetic parameters dependency on the mean mRNA expression (A) Burst size b in function of the mean mRNA expression $\langle m \rangle$. The range of expression covers by the clones is essentially explained by the burst size. (B) Cycle completion time $\tau_a + T$ in function of the mean mRNA expression $\langle m \rangle$. The cycle completion time is rather insensitive to $\langle m \rangle$ in our clones.

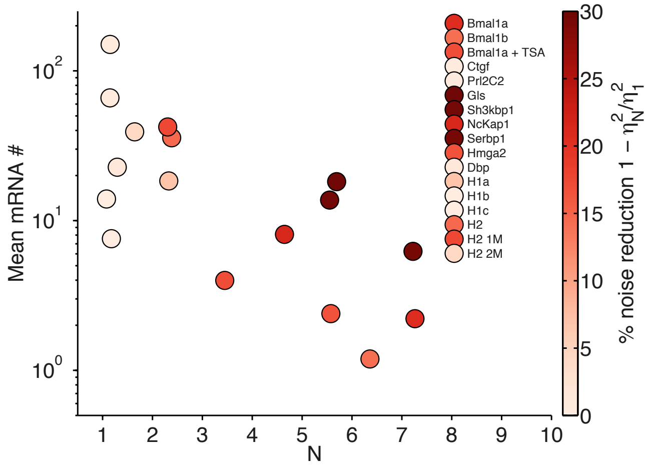

Figure S7: Noise reduction provided by the cycle compared to a simple switch (N=1), in function mean mRNA expression $\langle m \rangle$ and the mean number of inactive steps N. The genes benefiting the most from the noise reduction provided by the cycle are the ones expressed highly enough such that the promoter cycle dominates ($\eta_p^2 \ll \eta_c^2$) and exhibiting a large number of steps ($N \sim 6$). Even for theses genes, the actual noise reduction is at best 30%, which is approximately half the maximal theoretical reduction of 50% (for $N \to \infty$).

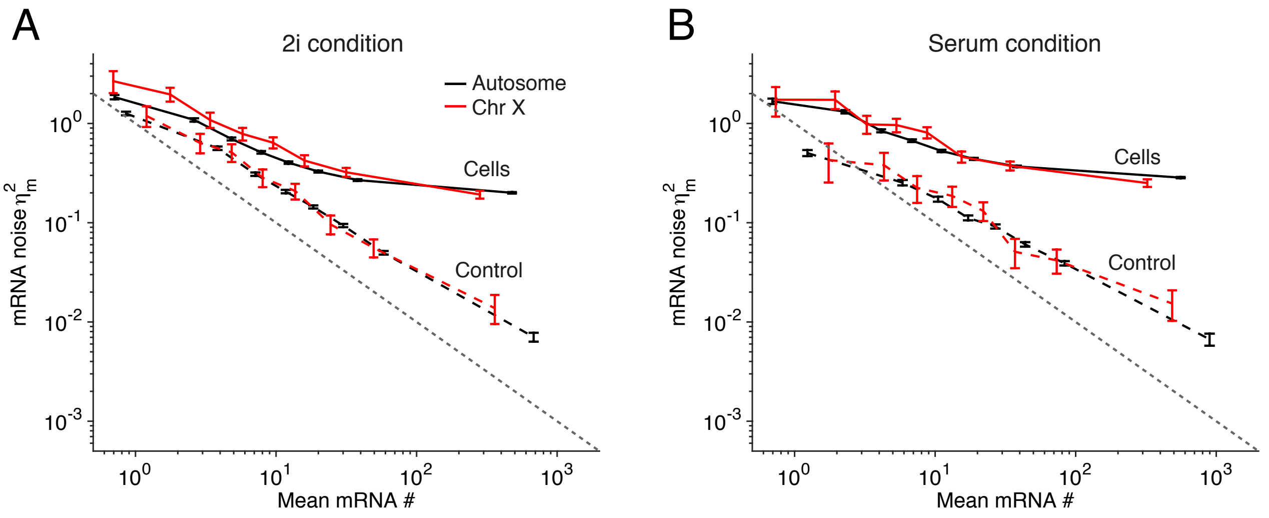

Figure S8: Biological noise inferred from single-cell RNA-seq in mouse embryonic stem cells. (A-B) Biological noise inferred with $\langle q \rangle = 0.1$ for autosomal (black) and chromosome X genes in both 2i (A) and serum conditions (B). The dash lines stand for the control (cells pooled together and split in single-cell equivalent) while the solid lines correspond to real cells. The error bars corresponds to 5 and 95th percentiles of the posterior distributions.

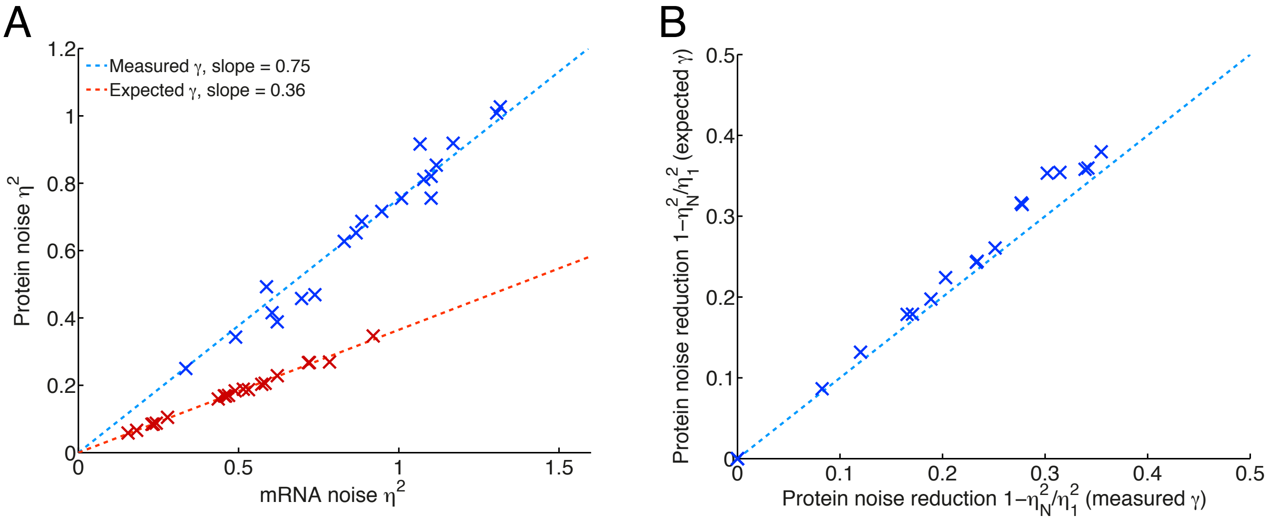

Figure S9: Propagation of transcriptional noise at the protein level. (A) Protein noise in function of transcriptional noise for the full set of clones. The blue crosses correspond to noise values computed using the experimentally measured decay time $\tau_p \sim 30$ min and $\tau_m \sim 100$ min leading to $\tau_m/(\tau_p + \tau_m) = 0.74$ on average, whereas the red crosses stand for noise values computed using more realistic values ($\tau_p = 5$ h and $\tau_m = 3$ h) giving $\tau_m/(\tau_p + \tau_m) = 0.37$. It follows that the slopes of the linear regressions closely match the factors $\tau_m/(\tau_p + \tau_m)$, confirming the noise propagation predicted by equation (Eq. 22). (B) Protein noise reduction provided by the number of inactive steps N, for the full set of clones. The noise reduction is almost unaffected by the corrected values of τ_m and τ_p, as indicated by the small deviations from dashed line of slope 1.

2 Real-time measurements of transcription in mammals

The single cell time-lapse bioluminescence traces analyzed in this study are taken from (Suter *et al.* , 2011). In addition, we generated new cell lines carrying a synthetic promoter using the Flp-In system. Here, we briefly review the experimental setup which was established in order to monitor transcription at high temporal resolution and the experimental procedures to generate new data.

2.1 Suter et al. data

A short-lived nuclear luciferase reporter (NLS-luc) encoded by a short-lived mRNA was integrated into three types of constructs:

i. A gene trap lentivector, allowing transcription to be controlled by the endogenous locus at the insertion site.
ii. Vectors conferring circadian expression by the *Bmal1* promoter.
iii. Vectors carrying artificial promoters consisting of one (*H1*) or two copies (*H2*) of different CCAAT box variants followed by a TATA box.

These constructs were used to generate monoclonal NIH-3T3 cell lines (mice fibroblasts). Non-dividing confluent cells were used during the recording to minimize noise from cell-cycle and avoid cell tracking issues (Zopf *et al.* , 2013). Images were captured every 5 minutes at single-cell resolution with a highly sensitive EMCCD camera during approximately 2 days. Calibration of the system was performed in order to relate amount of emitted light and the protein copy number. The probability of measuring the signal s given p proteins is then given by a normal distribution:

$$P_e(s|p) \sim \mathcal{N}(s; \alpha p, \sigma_b^2 + \beta p) \tag{1}$$

with the following parameters $\alpha = 7.63$, $\beta = 54$ and $\sigma_b \simeq 100$. In this study, we analyzed 16 different cell lines, 7 with different endogenous promoter, 2 with the *Bmal1* promoter, 1 with the *Dbp* promoter and 6 with synthetic promoters (among which *H1b* and *H1c* were newly generated, see below). The complete list of analyzed genes is given in Table EV1. Of note, we did not include in our analysis several clones from Suter *et al.* (2011), namely *Plectin1*, *H1 1M*, *H1 2M*, *H1 3M* and *H2 3M*, since the strength of the signal was not sufficient to resolve the structure of the promoter cycle accurately, as determined by simulation (cf. Section 5).

2.2 New data

Generation of additional Flp-In host cell lines To obtain cell lines carrying the *H1* construct inserted at different genomic locations, we first generated a collection of NIH-3T3 Flp-In host cell lines. To this aim, NIH-3T3 cells were plated in 6 well plates, and each well was transfected with 50ng of a modified version of the pFRT/lacZeo plasmid (Invitrogen), in which the sequence coding

for Zeocin resistance was replaced by the Neo coding sequence (plasmid kindly provided by Dr. Jürgen Ripperger). 48 hours after transfection, cells of individual wells were transferred into 15cm dishes and selected with G418 (Life Technologies) at a final concentration of 500 μg/ml. After 14 days, individual clones were picked and further expanded while maintaining the antibiotic selection.

Checking for single insertion of FRT site by Southern blotting To verify the presence of a single FRT site per clone, we first extracted genomic DNA from $4 \cdot 10^6$ cells per clone using GenElute Mammalian Genomic DNA Miniprep Kit (Sigma). 10 μg of genomic DNA per clone were then used to perform a Southern blot. The HindIII digestion of genomic DNA, electrophoresis, transfer and crosslink to a membrane, probe labeling using ^{32}P random priming and hybridization were performed by Lofstrand Labs Limited. The probe used to identify FRT inserts was generated from a 700 bp PCR fragment corresponding to a fraction of the neo gene. Two clones containing a single FRT site at different genomic locations were selected and used to generate Flp-In *H1* cell lines (clones *H1b* and *H1c*).

Generation of additional Flp-In H1 cell lines Clones *H1b* and *H1c* were co-transfected with pOG44 and a pcDNA5/FRT plasmid carrying the *H1* synthetic promoter driving the expression of the short-lived NLS-luc (Suter *et al.* , 2011). Cells were first cultured at 30°C for 48h, and transferred into 15cm dishes. 24h later, the medium was supplemented with hygromycin B (InvivoGen) at a final concentration of 200 μg/ml. After 20 days, individual clones were pooled and expanded in selective medium.

Time-lapse luminescence microscopy Time-lapse luminescence microscopy was performed as previously described (Suter *et al.* , 2011). Briefly, Flp-In *H1* cell were diluted 1:50 into non-luminescence NIH-3T3 cells, and plated in a 2.2cm glass bottom dish (WillCo) to a final amount of $9 \cdot 10^5$ per dish one day prior to recording. The following day, the medium was replaced by high-glucose DMEM without phenol red (Gibco) supplemented with 10% FBS (Sigma), 1% L-Glutamine-Penicillin-Streptomycin solution (Sigma) and D-luciferin (Life Technologies) at a final concentration of 100μM. Luminescence monitoring was performed in a LuminoView LV200 microscope (Olympus) with a cooled C9100-13 EM-CCD camera (Hamamatsu). During luminescence recording, cells were maintained at 37°C in a humid environment with 5% CO_2. Images were acquired with an exposure time and time resolution of 5 minutes during 48h. Recording of the luminescence signal was optimized by using a binning of 4, an EM gain of 150 and a 5x photon imaging mode. The time-lapse recordings were then analyzed using the ImageJ software by manual tracking individual cells in an area of 20 pixels over the entire movie.

3 Stochastic modeling of mammalian gene expression

In this section, we first present a class of nested stochastic promoter cycle models, which extend the standard Telegraph model (Peccoud & Ycart, 1995). We then show how we analyze the bioluminescence time traces of the different by computing the likelihood of the traces conditioned on a given promoter cycle model.

3.1 Extension of the Telegraph model: the promoter cycle

The simplest extension of the Telegraph model accounting for the observed refractory period in gene reactivation (Suter *et al.* , 2011; Harper *et al.* , 2011) is the proposed sequential inactive gene states model displayed in Figure 1A. In this kinetic model, the promoter transitions are modeled as an irreversible cycle formed by one active state (on-state) in which transcription can occur and N sequential inactive states (off-states). The proposed gene model is a simplification of the promoter progression model analyzed by Zhang et al. (Zhang *et al.* , 2012) as we only consider fully irreversible transitions between gene states, which reduces the number of kinetic parameters. Although more general distributions of silent periods could be obtained by including backward transitions (reversible reactions) or shortcuts to the active state in the promoter cycle, these sophistications are not required to capture the unimodal (peaked) property of the measured distributions, the key characteristic of the refractory period (Bel *et al.* , 2010).

The number of inactive states N defines a class of nested promoter models, which include the simple promoter switch of the Telegraph model as the case $N = 1$. To account for a refractory period, at least $N = 2$ sequential inactive states are necessary. Provided that the transition rates between the different inactive states have similar magnitudes, an increase in N will result in a more peaked distribution of silent periods. Importantly, the distribution only retain the signature of the slowest transitions (rate-limiting transitions), significantly faster transition rates are overshadowed by the rate-limiting ones.

In addition to the promoter cycle models, mRNA and protein copy numbers are modeled as birth-death processes. The resulting models are parametrized by $N+5$ constant rates: the promoter transition rates k_a, k_1,...,k_N, the transcription rate k_m, the translation rate k_p and the mRNA and protein degradation rates γ_p and γ_m. Among these kinetic rates, three were measured experimentally (Suter *et al.* , 2011), namely the translation rate k_p and the two degradation rates γ_p and γ_m which are given in Table EV1, while the other are unknown.

3.2 Likelihood calculations

To select the optimal promoter model and estimate the kinetic parameters for the different clones, we extended the computational method that we developed previously (Suter *et al.* , 2011). As a first step, we computed the probability that the time traces of luciferase activity were generated by our

gene expression models. Taking into account the Markovian nature of the models, the likelihood of a bioluminescence time trace $D = \{s_i\}$, given the number of inactive states N and the set of kinetic parameters θ_N, can be written as

$$\mathcal{L}(D|N,\theta_N) = \sum_{\{\Lambda\}} \prod_{i=1}^{L} P_e(s_i|p_i) P_t(p_i m_i g_i | p_{i-1} m_{i-1} g_{i-1}, N, \theta_N) P_e(s_0|p_0) P_s(p_0 m_0 g_0 | N, \theta_N) \qquad (2)$$

where $P_e(s_i|p_i)$ is the probability of observing the signal s_i given the amount of protein p_i at time i and $P_t(p_i m_i g_i | p_{i-1} m_{i-1} g_{i-1}, N, \theta_N)$ is the transition probability, *i.e.* the probability that the cell went from p_{i-1} proteins, m_{i-1} mRNA copies and gene state g_{i-1} at time $i-1$ to a new state $p_i m_i g_i$ at time i. The cells are assumed to be at steady-state, thus the initial condition $P_s(p_0 m_0 g_0 | N, \theta_N)$ is set as the stationary distribution of the system. The sum in equation (Eq. 2) runs over all hidden (unobserved) state trajectories $\Lambda = \{p_i m_i g_i\}$.

3.2.1 Transition probabilities

As shown in the previous section, computing the likelihood $\mathcal{L}(D|N,\theta_N)$ requires the transition probabilities of the system (the propagator) which in principle can be derived from the master equation of the model. However, it is often not possible to obtain an exact expression of the transition probabilities since solving the full master equation of the system is usually intractable. Despite this fact, the complexity of the master equation can be significantly reduced by making judicious assumptions. Indeed, the transition probability after time Δt can be computed by factorizing the full propagator:

$$P_t(pmg|p'm'g') \simeq P_t(p|p'm') P_t(mg|m'g') \qquad (3)$$

This factorization assumes that the amount of mRNA m does not change much during Δt which requires $\gamma_m \Delta t \ll 1$ (this is fulfilled in practice since $\gamma_m \simeq 0.01$ min^{-1} and $\Delta t = 5$ min). It is worth pointing out that we did not further factorize $P_t(mg|m'g')$ as done in our previous work (Suter *et al.* , 2011), therefore achieving a better approximation for fast gene transitions.

The protein dynamics is then described by a birth-death process whose master equation can be solved analytically leading to the following propagator:

$$P_t(p|p'm') = \sum_{q=0}^{p'} \binom{p'}{q} \mathcal{P}\left(p-q;\lambda\right) (e^{-\gamma_p \Delta t})^q (1-e^{-\gamma_p \Delta t})^{p'-q}$$

with $\mathcal{P}$ the Poisson distribution with parameter $\lambda = \frac{k_p m'}{\gamma_p}\left(1-e^{-\gamma_p \Delta t}\right)$. The mean and the variance

of $P_t(p|p'm')$ are given by

$$\mu_p(p', m', \Delta t) = \frac{k_p m'}{\gamma_p}\left(1 - e^{-\gamma_p \Delta t}\right) + p' e^{-\gamma_p \Delta t}$$
$$\sigma_p^2(p', m', \Delta t) = \frac{k_p m'}{\gamma_p}\left(1 - e^{-\gamma_p \Delta t}\right) + p' e^{-\gamma_p \Delta t}\left(1 - e^{-\gamma_p \Delta t}\right) \tag{4}$$

For a large amount of proteins, the transition probabilities were computed using the linear noise approximation. Specifically, when $2\sigma_p < \mu_p$,

$$P_t(p|p'm') = \frac{1}{\sqrt{2\pi\sigma_p^2(p', m', \Delta t)}} \exp\left(-\frac{(p - \mu_p(p', m', \Delta t))^2}{2\sigma_p^2(p', m', \Delta t)}\right)$$

The transition probabilities of the mRNA's and the gene $P_t(mg|m'g')$ are obtained from the following master equation describing the transcriptional dynamics of the promoter cycle:

$$\begin{aligned}\frac{d}{dt}P(m, g|t) = {} & k_m \delta_{g,0} P(m-1, g|t) - k_m \delta_{g,0} P(m, g|t) \\ & + (m+1)\gamma_m P(m+1, g|t) - m\gamma_m P(m, g|t) \\ & + k_g P(m, g-1|t) - k_{g+1} P(m, g|t)\end{aligned} \tag{5}$$

with $g = 0$ corresponding to the active state and the following periodic conditions $g = -1 \equiv N$ and $g = N + 1 \equiv 0$. After appropriate truncation on the mRNA copy number (Munsky & Khammash, 2006), the master equation can be written in term of a matrix L containing the propensity functions of the different reactions:

$$\frac{d}{dt}P(mg) = LP(mg) \tag{6}$$

where L can be decomposed as sum of tensor products of different matrices:

$$L = I_M \otimes K_N + G_M \otimes I_N + K_M \otimes R_N$$

with I_M and I_N standing for the identity matrix of size $M+1$ and $N+1$ where M is the maximum number of mRNA states after truncation and N the total number of inactive states of the promoter cycle. The matrix K_N encodes the rates of the possible transitions of the promoter cycle and is given as follows:

$$K_N = \begin{pmatrix} -k_1 & 0 & \dots & 0 & k_a \\ k_1 & -k_2 & \ddots & \vdots & 0 \\ 0 & k_2 & \ddots & 0 & \vdots \\ \vdots & \ddots & \ddots & -k_N & 0 \\ 0 & \dots & 0 & k_N & -k_a \end{pmatrix} \tag{7}$$

whereas G_M and K_M describe the degradation and production of mRNA,

$$G_M = \begin{pmatrix} 0 & \gamma_m & 0 & \dots & 0 \\ 0 & -\gamma_m & 2\gamma_m & \ddots & \vdots \\ 0 & 0 & -2\gamma_m & \ddots & 0 \\ \vdots & \ddots & \ddots & \ddots & M\gamma_m \\ 0 & \dots & \dots & 0 & -M\gamma_m \end{pmatrix} \qquad K_M = \begin{pmatrix} -k_m & 0 & \dots & \dots & 0 \\ k_m & -k_m & \ddots & \ddots & \vdots \\ 0 & k_m & \ddots & \ddots & \vdots \\ \vdots & \ddots & \ddots & -k_m & 0 \\ 0 & \dots & 0 & k_m & -k_m \end{pmatrix}$$

R_N indicates in which promoter state transcription occurs, in our model $R_N(n, n') = 1$ if $n = n' = 1$ and zero otherwise. The propagator of the resulting finite system (Eq. 6) can be expressed as a matrix exponential of the L operator:

$$P_t(mg|m'g') = \exp(L\Delta t) \tag{8}$$

In order to ensure proper normalization of the propagator, we closed the system by imposing $K_M(M+1, M+1) = 0$ which prevents probability leakage. In practice, such approximation proved to be convenient when setting a cutoff on the transition probabilities based on predefined percentiles. In addition, we ensure that M was large enough to capture the most probable transitions for each luminescent time trace.

3.2.2 Efficient likelihood computation

An efficient computation of the likelihood (Eq. 2) is critical since model selection and parameters estimation usually involve many evaluation of this function. The standard method to compute this quantity efficiently is the forward algorithm (Rabiner, 1989; Durbin, 1998). It introduces the so-called forward score $F_i(pmg)$ which is defined as the probability of the data up to time i and being, at that time, in state pmg. This quantity can be computed recursively using the following expression:

$$F_{i+1}(pmg) = P_e(s_{i+1}|p) \sum_{p'm'g'} P_t(p|p'm')P_t(mg|m'g')F_i(p'm'g') \tag{9}$$

and the probability of the data is then,

$$\mathcal{L}(D|N, \theta_N) = \sum_{pmg} F_L(pmg)$$

The core of the forward algorithm (Eq. 9) is nothing more than a sparse matrix vector multiplication provided some cutoff have been applied on the transition probabilities. Regarding the implementation, we took advantage of the factorization of the probability transitions (Eq. 3) in order to reduce the number of nested loops (Algorithm 1). We performed standard code optimization (C++ implementation), using contiguous data structure, data structure alignment and forcing

vectorization of the inner loops using SIMD instructions.

Algorithm 1 Forward Kernel

```
F_{i+1}(pmg) ← 0 ∀pmg
for all m'g' ∈ {mg}_i do
    G_{m'g'}(p) ← 0 ∀p
    for all p' ∈ {p}_i do
        for all p ∈ {p}_{i+1} do
            G_{m'g'}(p) ← G_{m'g'}(p) + P_t(p|p'm')F_i(p'm'g')
        end for
    end for
    for all p ∈ {p}_{i+1} do
        H_{m'g'} ← P_e(s_{i+1}|p)G_{m'g'}(p)
        for all mg ∈ {mg}_{i+1} do
            F_{i+1}(pmg) ← F_{i+1}(pmg) + P_t(mg|m'g')H_{m'g'}
        end for
    end for
end for
```

The transition probabilities of the protein $P_t(p|p'm')$ were computed on the fly and stored in order to avoid computation of unnecessary transitions and to minimize memory usage. In addition, to reduce number of indices for clones with large number of proteins, we aggregated the proteins in groups of $b_1, b_2, ...$ states using a linear aggregation operator A (Kemeny & Snell, 1976), where the size of the groups were based on the standard deviation of the protein transition probabilities (Eq. 4).

$$A = \begin{pmatrix} \overbrace{1...1}^{b_1} & 0...0 & \cdots \\ 0...0 & \overbrace{1...1}^{b_2} & \cdots \\ \vdots & \vdots & \ddots \end{pmatrix}$$

Therefore the protein transition probabilities in the aggregated space $\{\bar{p}\}$ are then given by

$$\bar{P}_t(\bar{p}|\bar{p}'m') = AP_t(p|p'm')D$$

where D is the disaggregation operator defined as the Moore-Penrose pseudoinverse of A.

Since the full likelihood is essentially the product of the likelihood of each cell time trace, i.e. $\mathcal{L}(D|N, \theta_N) = \prod_{i=1}^{N_{cell}} \mathcal{L}(D_i|N, \theta_N)$, the algorithm has a natural parallel structure which can be exploited. Because the transition probabilities for mRNAs and the gene (Eq. 8) were shared among the different cells, each cell (core) only computed a block of the transition matrix $P_t(mg|m'g')$ using the Krylov-based algorithm of Expokit (Sidje, 1998), the final matrix is then reassembled from the different blocks thanks to message passing interface (MPI).

4 Model selection and parameters estimation

According to the standard Bayesian paradigm, we based the whole inference, namely the model selection and the parameters estimation, on the joint posterior distribution:

$$P(N, \theta_N | D) = \frac{\mathcal{L}(D|N, \theta_N) P(N, \theta_N)}{\sum_N \int \mathcal{L}(D|N, \theta_N) P(N, \theta_N) d\theta_N} \tag{10}$$

where N is the number of inactive steps, θ_N the parameters of the model, D the bioluminescence time traces, $\mathcal{L}(D|N, \theta_N)$ the likelihood of the data and $P(N, \theta_N)$ the prior distribution. We sampled the joint posterior distribution $P(N, \theta_N|S)$ with the Reversible Jump Markov-Chain Monte Carlo (RJ-MCMC) algorithm (Green & Hastie, 2009).

In the following section, we first define a suitable parametrization for the sampling, then we express the prior distribution in that parameter space and finally we detail the RJ-MCMC scheme we implemented to estimate the number and the timescales of the rate-limiting steps underlying the silent period of the different promoters.

4.1 Parametrization of the kinetic model

The unknown kinetic parameters of the model were: k_m, k_a, k_1, k_2, k_3, ..., k_N, which are the transcription rate, and the different rate of transition between the promoter states respectively. The mean active time is given by $\tau_a = 1/k_1$, and the mean time spent in each inactive state by $\tau_i = 1/k_{i+1}$ for $i < N$ and $\tau_N = 1/k_a$.

As we will see later, a parametrization which proved to be more convenient, can be expressed in terms of the following independent parameters: b, τ_a, T, p_1, p_2, ..., p_{N-1} where $b = k_m \tau_a$ is the burst size, $T = \sum_{i=1}^{N} \tau_i$ the mean total silent period and $p_i = \tau_i / T$ the fraction of time spent in the ith inactive state. Consequently, p_N is determined by the constraint $\sum_{i=1}^{N} p_i = 1$ and it follows that the $\{p_i\}$ lie on the $(N-1)$-simplex.

4.1.1 Symmetry of the promoter cycle

There is an inherent symmetry in our gene expression models, namely the likelihood function $\mathcal{L}(D|N, \Theta_N)$ is invariant under permutations of the different inactive steps. This property is a direct consequence of the considered promoter cycles since mRNA transcription can only occur when the gene is in the active state. Thus, we can only expect to reconstruct the distribution of active and silent periods, and not the exact sequence of the inactive steps. Indeed, the distribution of silent periods is given by the convolution of the distributions of each inactive step, and since the convolution is symmetric, the ordering of the inactive steps cannot be resolved. However, the shape of the distribution does depend on the time scales of the different inactive steps and therefore, in principle, these can be inferred from the data.

4.1.2 Parameterization of the cycle

To take into account the symmetry previously mentioned, we design an adequate parametrization. In practice, we restrict the space of the simplex to a subspace for which the p_i satisfy an ordering condition $p_1 \geq p_2 \geq ... \geq p_N$. This can be achieved using the following parametrization:

$$\begin{cases} p_2 = u_1 p_1 \\ p_3 = u_2 p_2 \\ \quad ... \\ p_N = u_{N-1} p_{N-1} \end{cases} \tag{11}$$

where $u_i \in [0, 1]$. Recalling that p_N is given by the constraint $\sum_{i=1}^{N} p_i = 1$, we have actually the same number $(N-1)$ of independent parameters u_i than p_i.

Change of variables Using the constraint $\sum_{i=1}^{N} p_i = 1$ and the relation (Eq. 11), we can find the explicit change of variables from the u space to the p space. First, we can express p_1 in terms of the u_i by solving the following system of equations:

$$\begin{cases} p_N = 1 - \sum_{i=1}^{N-1} p_i = 1 - p_1 \left(1 + \sum_{j=1}^{N-2} \prod_{i=1}^{j} u_i \right) \\ p_N = p_1 \prod_{i=1}^{N-1} u_i \end{cases}$$

We find that

$$p_1 = \frac{1}{1 + \sum_{j=1}^{N-1} \prod_{i=1}^{j} u_i}$$

and consequently for $k > 1$ using relation (Eq. 11), we finally obtain

$$p_k = \frac{\prod_{i=1}^{k-1} u_i}{1 + \sum_{j=1}^{N-1} \prod_{i=1}^{j} u_i}$$

Jacobian Having specified the transformation $p \to u$, we computed the Jacobian which is required to express the prior in the u space given a prior in the p space:

$$P(u_1, ..., u_{N-1}) \mathrm{d}u_1 \cdot ... \cdot \mathrm{d}u_{N-1} = P(p_1, ..., p_{N-1}) \mathcal{J}_{N-1} \mathrm{d}u_1 \cdot ... \cdot \mathrm{d}u_{N-1}$$
$$\text{with} \quad \mathcal{J}_{N-1} = \left| \frac{\partial(p_1, ..., p_{N-1})}{\partial(u_1, ..., u_{N-1})} \right|$$

In term of the u_i, the Jacobian $\mathcal{J}_{N-1}$ has the following form:

$$\mathcal{J}_{N-1} = \frac{\prod_{i=1}^{N-2} u_i^{N-1-i}}{\left(1 + \sum_{j=1}^{N-1} \prod_{i=1}^{j} u_i\right)^N} \tag{12}$$

4.2 Prior distributions

As required in Bayesian inference, we have to define a prior distribution which encodes our initial state of knowledge concerning the models and the parameters. A natural choice was to factorize it in the following manner:

$$P(N, \theta_N) = P(\tau_a) P(T) P(b) P(u_1, ..., u_{N-1}) P(N|T) \tag{13}$$

In the following subsection we will specify the different terms in the right hand side of the expression above (Eq. 13).

4.2.1 Priors on kinetic parameters

Recalling that the active time τ_a and the total silent period T are real positive parameters, the corresponding uninformative prior is log-uniform. Although, it is possible to resolve small timescales providing sufficient amount of data (structural identifiability), in practice the likelihood function can be rather insensitive to small timescale variations (when $\tau \ll \Delta t$). In order to restrict the parameters space to resolvable scales, we added a penalization to the log-uniform prior based on the sampling time Δt:

$$P(\tau) = \frac{1}{\tau} \exp\left(-\frac{\Delta t}{\tau}\right)$$

This prior was used for τ_a and T and its improper nature did not represent a problem for the inference since these parameters are shared among the different models and the resulting posteriors were normalizable.

The prior for the burst size b was set by constraining the transcription rate k_m. Assuming that the effective transcription rate is bounded, a suitable uninformative prior is a bounded uniform

distribution. In terms of burst size this condition leads to

$$P(b) = 1/\tau_a$$

Finally, the prior on the $\{u_i\}$ was defined with respect to the $\{p_i\}$, we set a uniform prior on the (N-1)-simplex. Thanks to the Jacobian of the coordinate transform (Eq. 12), the resulting normalized prior in u-space is given by:

$$P(u_1, u_2, ..., u_{N-1}) = (N-1)!N! \frac{\prod_{i=1}^{N-2} u_i^{N-1-i}}{\left(1 + \sum_{j=1}^{N-1} \prod_{i=1}^{j} u_i\right)^N}$$

where the factor $(N-1)!N!$ stands for the volume normalization of the (N-1)-simplex and the $N!$ symmetric sectors.

4.2.2 Prior on the number of inactive steps

We designed a prior for the number of inactive steps, in order to penalize models which lead to unrealistically small timescale for the $\{\tau_i\}$, namely timescales which are smaller than the sampling time Δt of 5 min. In term of the $\{p_i\}$, since p_N is the smallest fraction due to the ordering condition, this can be stated as $p_N > \lambda$ where $\lambda = \tau_t/T$ with τ_t the time threshold. With help of geometric arguments, we can express the volume reduction of the simplex space implied by that condition. We found that the new volume of one symmetric sector S'_{N-1} is given by

$$S'_{N-1} = (1 - N\lambda)^{N-1} S_{N-1}$$

where S_{N-1} is the volume of the sector without the constraint on p_N. The ratio S'_{N-1}/S_{N-1} can be viewed as a probability based on the available volume when $p_N > \lambda$. Normalizing this quantity over $N \in \mathbb{N}^*$ and setting $\tau_t = \Delta t$ leads to the following prior for the number of inactive steps:

$$P(N|T) = \begin{cases} \frac{1}{Z}\left(1 - N\frac{\Delta t}{T}\right)^{N-1} & \text{if } N \leq N_{max} = \min\left\{1, \left\lfloor \frac{T}{\Delta t} \right\rfloor\right\} \\ 0 & \text{otherwise} \end{cases}$$

where Z is the normalization factor:

$$Z = \sum_{n=1}^{N_{max}} \left(1 - n\frac{\Delta t}{T}\right)^{n-1}$$

4.3 Reversible Jump MCMC

The Reversible Jump MCMC algorithm extends the scope of the well-known Metropolis-Hastings (M-H) algorithm (Green & Hastie, 2009; Andrieu *et al.* , 2003). In essence RJ-MCMC is very similar except that the sampling space $\mathcal{X} = \bigcup_{N\in\mathcal{M}}(\{N\} \times \Theta_N)$ usually involves parameter spaces Θ_N of different dimensions. In order to build a Markov chain on this space with the desired limiting distribution, *i.e.* the joint posterior distribution $P(N, \theta_N|D)$, we first need to define suitable mappings between the different model parameters spaces Θ_N, task which is carried out by introducing auxiliary variables r_N to ensure dimension matching between the spaces. Specifically, we have to define a diffeomorphism $H_{N'\to N}$ for every different moves $N' \to N$ for which $N' \neq N$ such that

$$H_{N'\to N}(\theta_{N'}, r_{N'}) = (\theta_N, r_N)$$
$$\dim(\theta_{N'}) + \dim(r_{N'}) = \dim(\theta_N) + \dim(r_N)$$

Using these mappings, across-model proposal distributions $Q_{N'\to N}$ are built to "jump" from one model to another. By imposing reversibility of the chain (detailed balance) as in standard MCMC, the acceptance probability $\mathcal{A}_{N'\to N}$ of such a move can be specified to ensure convergence toward the desired posterior distribution.

In practice, RJ-MCMC works as a hybrid sampler, depending on the proposed move $N' \to N$, either a within-model M-H step is performed if $N' = N$, or otherwise an across-model jump according to the mapping $H_{N'\to N}$. The iterative protocol to sample from the joint posterior distribution is then quite simple:

1. Choose a move $N' \to N$ for the chain randomly
2. Generate new parameters (N, θ_N) according to the proposal distribution
3. Compute the likelihood $\mathcal{L}(D|N, \theta_N)$ for the proposed parameters
4. Sample α from a uniform distribution $\mathcal{U}_{[0,1]}$. If $\alpha < \mathcal{A}_{N'\to N}$, then accept the move and update the chain
5. Repeat the process, go back to 1.

where the acceptance probability $\mathcal{A}_{N'\to N}$ of the move $N' \to N$ is defined as follows:

$$\mathcal{A}_{N'\to N} = \begin{cases} \text{if } N' = N & \min\left\{1, \dfrac{\mathcal{L}(D|N,\theta_N)P(N,\theta_N)Q(\theta'_N|\theta_N)}{\mathcal{L}(D|N,\theta'_N)P(N,\theta'_N)Q(\theta_N|\theta'_N)}\right\} \\ \text{if } N' \neq N & \min\left\{1, \dfrac{\mathcal{L}(D|N,\theta_N)P(N,\theta_N)Q(N'|N)Q_{N\to N'}(r_N|\theta_N)}{\mathcal{L}(D|N',\theta_{N'})P(N',\theta_{N'})Q(N|N')Q_{N'\to N}(r_{N'}|\theta_{N'})}\mathcal{J}_{H_{N'\to N}}\right\} \end{cases}$$

This quantity is expressed in terms of the M-H proposal distribution of the parameters $Q(\theta_N|\theta'_N)$, the proposal distribution of the model $Q(N|N')$, the proposal distribution of the auxiliary variables

$Q_{N'\to N}(r_{N'}|\theta_{N'})$ and the Jacobian of the mapping:

$$\mathcal{J}_{H_{N'\to N}} = \left|\frac{\partial(\theta_N, r_N)}{\partial(\theta_{N'}, r_{N'})}\right| = \left|\frac{\partial H_{N'\to N}(\theta_{N'}, r_{N'})}{\partial(\theta_{N'}, r_{N'})}\right|$$

4.3.1 Move sets & proposal distributions

Since the choice of the move set, the mappings and the proposal distributions are problem-dependent, we detail in the following paragraphs our problem specific implementation. We introduced three different types of moves in order to sample the posterior distribution of the kinetic parameters and the number of inactive steps N in the cycle. The first is a within-model move, namely a M-H move which keeps the number of inactive steps constant ($N \to N$) and update the kinetic parameters according to the proposal distribution. Two types of across-model moves are required to change the dimensionality of the parameters space, namely forward moves which add a short inactive step in the cycle ($N \to N+1$) and backward moves which remove the shortest inactive step ($N \to N-1$). These two types of moves operate such that the burst size b, the active time τ_a and the total inactive time T is kept constant, facilitating the transition between the different models.

The resulting jump scheme is then quite simple, the chain explores the model space $\mathcal{M} = \{1, 2, ..., N_{max}\}$ by incrementing or decrementing by 1 the number of inactive steps N. By assigning equal probability to perform a forward or a backward move, the proposal distribution of the model $Q(N|N')$ can be expressed as a stochastic matrix:

$$Q(N|N') = \begin{pmatrix} 0 & 1/2 & & & 0 \\ 1 & 0 & \ddots & & \\ & 1/2 & \ddots & 1/2 & \\ & & \ddots & 0 & 1 \\ 0 & & & 1/2 & 0 \end{pmatrix}$$

where we took into account the fact that there is only one available transition at the boundary i.e. $N' = 1$ or $N' = N_{max}$. Regarding the value of N_{max}, we set it to 10 and checked afterwards that the model space was large enough to encompass the inferred value from real data.

Within-model move: Although the kinetic parameters of the model are slightly correlated and a multivariate distribution would potentially lead to a more efficient sampling in term of mixing time of the chain, we experienced that factorizing the M-H proposal distribution in different components as follows “is good enough”:

$$Q(\theta_N|\theta'_N) = Q_s(b|b')Q_s(\tau_a|\tau'_a)Q_s(T|T')\prod_{i=1}^{N-1} Q_u(u_i|u'_i) \tag{14}$$

where the proposal distributions $Q_s(\theta|\theta')$ for the real positive parameters (b, τ_a, T) were set as log-normal $q_L(\theta; \log\theta', \sigma_\theta)$ with their own scale parameter σ_θ and the proposal distribution for the u's $Q_u(u|u')$ as beta distribution $q_B(u; \alpha(u'), \beta(u'))$ where $\alpha(u') = 1 + \lambda_N u'$ and $\beta(u') = 1 + \lambda_N(1 - u')$ with λ_N a scale parameter depending on N. The different scale parameters were tuned to achieve satisfactory acceptance of the moves.

Forward move: We defined the mapping $H_{N\to N+1}$, which determines the new fractions of the inactive time p_i in terms of the previous ones p'_i and corresponds to adding a short inactive step $p_{N+1} \leq p_N$ to the cycle such that the total inactive time T is conserved, as follows:

$$H_{N\to N+1}(p'_1, p'_2, ..., p'_N, r) = \begin{cases} p_1 = p'_1/s \\ p_2 = p'_2/s \\ \vdots \\ p_N = p'_N/s \\ p_{N+1} = rp'_N/s \end{cases}$$

where $r \in (0, 1]$ is an auxiliary variable and $s = 1 + rp'_N$ is a normalization factor. Using relation (Eq. 11), the corresponding mapping in the u-space is simply given by:

$$H_{N\to N+1}(u'_1, u'_2, ..., u'_{N-1}, r) = \begin{cases} u_1 = u'_1 \\ u_2 = u'_2 \\ \vdots \\ u_{N-1} = u'_{N-1} \\ u_N = r \end{cases}$$

It follows that the Jacobian $J_{N\to N+1}$ of the transformation is then trivial, $J_{N\to N+1} = 1$. The proposal distribution $Q_{N\to N+1}(r|\theta_N)$ associated with this mapping was set as a uniform distribution on the interval $(0, 1]$.

Backward move: The reverse mapping $H_{N+1\to N}$ which removes the smallest inactive step while keeping the total inactive time T constant, is defined as the inverse of $H_{N\to N+1}$. It follows that the resulting mapping in the u-space is then trivially given by:

$$H_{N+1\to N}(u'_1, u'_2, ..., u'_N) = \begin{cases} u_1 = u'_1 \\ u_2 = u'_2 \\ \vdots \\ u_{N-1} = u'_{N-1} \\ r = u'_N \end{cases}$$

where r is again an auxiliary variable. Here also, $J_{N+1\to N} = 1$, consistent with the forward move. In this case, the proposal distribution $Q_{N+1\to N} = 1$ since no auxiliary random variable is required.

4.3.2 Sampling procedure & error estimates

For every set of cells, we performed 10'000 iterations with a burn-in period of 500 to 600 iterations on average, which proved to be enough to start sampling from the joint posterior distribution. We reported the mean and the 5 and 95th percentiles of the marginal posterior distributions as the final estimates for the unknown parameters. We also computed the Monte Carlo standard error (MSE) for the different estimates using overlapping batch means (Flegal & Jones, 2010). The latter quantity provides a good assessment of the mixing properties of the chain. Indeed, computing the ratio between the Monte Carlo variance σ^2_{MC} and the sample variance σ^2 enables estimation of the serial correlations in the generated sample:

$$\frac{\sigma^2_{\text{MC}}}{\sigma^2} = 1 + 2\tau$$

where τ is the autocorrelation time. A small value for τ reflects good mixing of the chain, i.e. the smaller the autocorrelations time, the closer the sample will be from independently drawn random variables.

5 Performance

Given limited amount of data, determining the optimal number of inactive steps of the promoter cycle and the associated partition of the silent period is particularly challenging since the signature of multiple sequential inactive steps in the bioluminescence time traces is subtle. The information regarding the structure of the promoter cycle essentially lies in the shape of the distribution of silent periods which relies on accurate reconstruction of the gene activity pattern from the bioluminescence time traces.

Here, we present the performance of our inference procedure based on RJ-MCMC sampling. First, we show how the partition of the silent period can be reconstructed from the MCMC samples. We then aim to validate the method, by analyzing multiple synthetic data sets. We discuss the performance of the method and its limit, in conditions close to the experimental ones. Finally, we present the inferred kinetic parameters of the optimal promoter cycle model for the full set of clones.

5.1 Inferring the silent partition

In principle, the parameterization introduced earlier (Section 4.1.2) to address the structural identifiability of the cycle enables the inference of asymmetric partitions, however, it can introduce a bias for symmetric partitions. Indeed, since we restricted ourselves to ordered partitions ($p_1 \geq p_2 \geq ... \geq p_N$), *i.e.* we only sample one single symmetric sector of the $(N-1)$-simplex, the mean of the sample will be biased toward a decreasing sequence. For example, consider the extreme symmetric case of a flat posterior in the 2-simplex, while on the full space the mean is centered and trivially given by $p_1 = p_2 = p_3 = 1/3$, computing the mean on a single symmetric sector leads to a biased partition: $p_1 = 11/18$, $p_2 = 5/18$, $p_3 = 1/9$. The magnitude of the bias lessens as the symmetric posterior becomes more peaked.

Assuming that the true posterior distribution of the partition conditioned on the model, *i.e.* $P(p_1, p_2, ..., p_N|N, D)$, can be approximated by a Dirichlet distribution with parameters $\boldsymbol{\alpha}$. Due to the symmetry of the cycle, the effective posterior distribution is invariant under permutation of the $\{p_i\}$, and thus would be given by

$$P(p_1, p_2, ..., p_N|\boldsymbol{\alpha}) = \frac{1}{N!B(\boldsymbol{\alpha})} \sum_{\sigma \in S_N} \prod_{i=1}^{N} p_{\sigma_i}^{\alpha_i - 1} \tag{15}$$

where the sum runs over all permutations of the $\{p_i\}$ and $B(\boldsymbol{\alpha})$ is the multinomial Beta function. Such a distribution (Eq. 15) provides a way to estimate the mean of the true posterior, by maximizing the likelihood of the the MCMC sample according to $\boldsymbol{\alpha}$. Indeed, after reordering of the α_i, the unbiased mean partition is the given by $(\alpha_1, \alpha_2, ..., \alpha_N)/\sum_{i=1}^{N} \alpha_i$.

5.2 Validation with synthetic data

In order to assess the performance of the designed RJ-MCMC sampler, we generated various sets of synthetic data with the Gillespie algorithm (SSA) (Gillespie, 1977). We simulated luminescence time traces according to the microscope noise model (Eq. 1) and the stochastic promoter cycle model, using a sampling time $\Delta t = 5$ min for a total duration of 2 days, mimicking the experimental recording conditions (Suter *et al.* , 2011).

Toy examples: The first collection of simulated data was designed to illustrate the RJ-MCMC procedure. We generated 4 sets of 64 cells, with the following common parameters: $k_p = 0.2$ min^{-1}, $\gamma_p = 0.0318$ min^{-1}, $k_m = 5$ min^{-1}, $\gamma_m = 0.0102$ min^{-1}, $\tau_a = 8$ min and $T = 90$ min. The 4 sets differ only in term of number of inactive states N, namely the partition of the total inactive time T is different among them, the first set was generated with a unique inactive state ($\tau_1 = 90$ min), the second with two inactive states ($\tau_1 = 60$ and $\tau_2 = 30$ min), the third with 4 equal inactive states ($\tau_i = 22.5$ min for $i = 1, ..., 4$) and the last with 6 equal inactive states ($\tau_i = 15$ min for $i = 1, ..., 6$). One simulated cell of the first and last set are displayed in Figure 1B and 1C respectively.

The estimated parameters are given in Table EV2. The posterior distributions of the parameters for the different sets are displayed in Figure 2B and 2C. Overall, we recovered the input parameters with good accuracy. The number of inactive states tend to be slightly overestimated. The optimal number of inactive states for the second, third and fourth data set, defined as the mode of the posterior distribution were 3, 5 and 7 respectively, instead of 2, 4 and 6. Regarding the inferred partition of the total silent period, we recovered successfully the asymmetry of the partition for the second data set, whereas for the third and fourth data set the inferred partition was slightly biased toward a decreasing sequence instead of a fully symmetric partition. This effect was expected, as explained in Section 5.1. Correcting the bias introduced by the restriction of sampling within a single symmetric sector of the $(N-1)$-simplex, the estimated partition was then more regular for the third and fourth data set. Finally, we estimated the relative Monte Carlo standard error (RMSE) for the main parameters, the RMSE remained smaller than 1% for most parameters.

Controlled simulations: Here, we explored the limits of our inference framework, namely how the level of expression and short active time τ_a affects our inference capability. We generated 48 cells with different mean levels of mRNA expression $\langle m \rangle$, by increasing either the transcription rate k_m or both k_m and τ_a. The following parameters were common in every cell: $k_p = 0.6$ min^{-1}, $\gamma_p = 0.0318$ min^{-1}, $\gamma_m = 0.0100$ min^{-1}, $T = 57$ min and $N = 5$. In both scenarios, the mean level of expression $\langle m \rangle$ was used as a control parameter, ranging from 2.5 to 25 transcripts. In the first scenario we set τ_a fixed to 3 min, below the sampling time Δt of 5 min, and increased the

transcription rate according to $\langle m \rangle$ using the steady-state expression for the mean:

$$k_m = \gamma_m \frac{\tau_a + T}{\tau_a} \langle m \rangle$$

While in the second scenario, we increased both k_m and τ_a with the constraint that $\tau_a = 6k_m$. Using the steady-state expression for $\langle m \rangle$, the transcription rate is then simply given by:

$$k_m = \frac{1}{2}\gamma_m \langle m \rangle + \frac{1}{2}\sqrt{\gamma_m^2 \langle m \rangle^2 + \frac{2T}{3}\gamma_m \langle m \rangle}$$

The estimated parameters for the various conditions are given in Table EV2 and shown in Figure 2D. In both scenarios, all the parameters are satisfactorily recovered, even when the cells are lowly expressed or the active period is smaller than the sampling time.

Heterogeneity bias: Finally, we investigated the effect of heterogeneity in the data on the inferred parameters. This aspect is particularly relevant, even in isoclonal populations, as cells are expected to display heterogeneity due to external and internal environmental fluctuations in addition to the intrinsic stochasticity of gene expression. In practice, such heterogeneity could be modeled as cells having their own kinetic parameters, or alternatively the whole population could be described with common effective parameters. The latter approach, used here, is more straightforward and provides more precise estimates since all the cells contribute to the inference. However, the estimated effective parameters could be biased by not reflecting the mean parameters of the population.

In order to characterize the potential biases due to a global fitting on cell populations, we first generated two populations of 48 cells sharing similar kinetic parameter except the transcription rate which was set to $k_m^{(1)} = 1$ in the first population and to $k_m^{(2)} = 5$ in the second one, we then generated several subset of data resulting from the mixing of the two populations in controlled ratios. We defined a control parameter r reflecting the fraction of cells from the second population in the final mixed collection of 48 cells. The list of shared parameters were the following: $k_p = 0.2$ min^{-1}, $\gamma_p = 0.0318$ min^{-1}, $\gamma_m = 0.0102$, $\tau_a = 8$ min and $T = 90$ min.

The estimated parameters for the different values of r are given in Table EV2 and shown in Figure S1. We predicted the behavior of the inferred parameters based on the hypothesis that the mean mRNA level $\langle m \rangle$ of the mixed population is correctly inferred and that the estimated transcription rate is actually the maximum among the underlying populations. This hypothesis provides a good explanation for the observed behavior of the estimated parameters, as shown in Figure S1. Indeed, the inferred mean mRNA expression follows closely the mean expression of the mixed population (red dashed line), computed as the weighted average between the first and second

initial population:

$$\langle m \rangle = \frac{k_m(r)}{\gamma_m} \frac{\tau_a}{\tau_a + T} \quad \text{with} \quad k_m(r) = (1-r)k_m^{(1)} + rk_m^{(2)}$$

The inferred transcription rate stays stable around the maximum of the mixed population $k_m^{(2)} = 5$ as r decreases and finally collapses to $k_m^{(1)} = 1$ when $r = 0$. Consequently, the inferred active period τ_a should decrease with r to compensate the extra contribution to the mean expression, in fact the estimated τ_a closely follows (red dashed line):

$$\tau_a(r) = \frac{\tau_a T k_m(r)}{(\tau_a + T)k_m^{(2)} - \tau_a k_m(r)} \quad \text{for} \quad r > 0$$

which is the predicted behavior of τ_a if the aforementioned hypothesis holds true. Regarding the burst size and total inactive period T, these are only slightly overestimated for the mixed population whereas the number of inactive states N is unaffected. Thus, it appears that the inferred active period τ_a can be biased toward a lower value in heterogenous population, whereas the inferred transcription rate tends to reflect the maximum rate in the population. Not so surprisingly, the model with the maximum transcription rate is more flexible, as it can account for a larger range of transcription rates (slopes in the mRNA time traces) by modulating the active period or by successive short activations.

5.3 Parameters of each clone & optimal model

We then applied our inference framework on the time-lapse cell recordings of the different clonal populations. We used the experimentally measured parameters from Suter et al. (Suter *et al.* , 2011; Molina *et al.* , 2013) which are given in Table EV1 .

The estimated number of inactive states, the kinetic parameters and the partition of the silent period for the different clones are given in Table EV3 and shown in Figure 3A-D. The optimal model was defined from the mode of the posterior distribution of the number of inactive states. The fraction of time the gene is active is given by $f_a = \tau_a/(\tau_a + T)$ which is displayed in percent. In Figure 3A-B, we show the 95% confidence ellipses which were computed from the sample covariance of the joint posterior distribution of the burst size $b = k_m \tau_a$ and the active fraction f_a (Figure 3A), and between the total silent period T and the burst size b (Figure 3B). The corrected partition of the silent period (cf. Section 5.1) was used in Figure 3D.

6 Silent period, active period & steady-state distribution

In order to assess the consistency of the promoter cycle model with the data, we compared the distribution of silent periods, active periods and the mRNA steady-state distribution predicted by the optimal model (modeled distributions) with the distributions inferred from the bioluminescence time traces. For that purpose, we designed a Gibbs sampler which enables reconstruction of those distributions conditioned on the data, using the optimal model as a prior.

Below, we first review the Gibbs sampling approach, and then derived the theoretical distributions according to the model. Finally, we show the comparison between the inferred and modeled distributions for simulated cell populations and the full set of experimental clones.

6.1 Gibbs sampling

Once model selection and parameter estimation was achieved, we reconstructed the mRNA steady-state distribution as well as the distribution of silent periods conditioned on the data. This task requires the probability of a hidden states trajectory $\Lambda = \{p_i m_i g_i\}$ given the bioluminescence time traces and the parameters:

$$P(\Lambda|D, N, \theta_N) = \frac{P(D, \Lambda|N, \theta_N)}{\mathcal{L}(D|N, \theta_N)}$$

where $P(D, \Lambda|N, \theta_N)$ is nothing else than the likelihood (Eq. 2) without the marginalization over all trajectories Λ. We sampled the joint distribution $P(\Lambda|D, N, \theta_N)$ using Gibbs sampling (Gelfand & Smith, 1990). This MCMC variant allows sampling from the joint distribution by sampling iteratively each state $\lambda_i \equiv p_i m_i g_i$ from the conditional distribution:

$$P(\lambda_i|\Lambda_{-i}, D, N, \theta_N) = \frac{P(\Lambda|D, N, \theta_N)}{P(\Lambda_{-i}|D, N, \theta_N)} = \frac{P(\Lambda|D, N, \theta_N)}{\sum_{\lambda_i} P(\Lambda|D, N, \theta_N)}$$

This quantity is much easier to compute than the joint distribution $P(\Lambda|D, N, \theta_N)$. In our case it reduced to:

$$P(\lambda_i|\Lambda_{-i}, D, N, \theta_N) \propto P_t(p_{i+1}m_{i+1}g_{i+1}|p_i m_i g_i) P_e(s_i|p_i) P_t(p_i m_i g_i|p_{i-1}m_{i-1}g_{i-1})$$

In practice, at every iteration, we chose randomly a time point i and we resampled the state of the system λ_i from the conditional distribution above. We recorded a hidden state trajectory Λ every 8'000 iterations which ensures that every time point was chosen and resampled approximately 10 times on average. After having discarded the first 1'000 trajectories, we recorded the 2'000 subsequent ones.

For each cell, the distributions of silent periods, active periods and mRNAs were reconstructed from the sampled trajectories $\{\Lambda\}$. The first distribution was computed by counting the number of intervals of length $n\Delta t$ that occurred between two active states. Similarly, the distribution of

active periods was estimated by counting the number of intervals of length $n\Delta t$ in the active state. The mRNA distribution was obtained by counting the number of occurrence of m transcripts all along the trajectories. The final distributions for the different cell populations were reconstructed as a mixture of each cell distribution (weighted according to the length of the recorded signal time traces).

6.2 Modeled distribution of silent & active periods

The silent period of the promoter cycle, *i.e.* the time between two successive activations, formally follows a phase-type distribution which describes Poisson processes occurring sequentially or in parallel. Since in practice the temporal observations are discrete in time, with intervals given by the sampling time Δt, we could potentially miss activation events between two observations, provided the on-time is short enough compared to Δt. The discrete waiting time, which takes into account the missing events, can be computed as follows. Starting in $g = 1$ the first inactive state, we defined an initial vector F_0 of size $N + 1$:

$$F_0(g) = \begin{cases} 1 & \text{if } g = 1 \\ 0 & \text{otherwise} \end{cases}$$

and the following recursion scheme:

$$F_{i+1}(g) = \sum_{g' \neq 0} P_t(g|g')F_i(g')$$

where the sum runs over all promoter states except the active one $g = 0$ and $P_t(g|g')$ the transition probabilities of the gene expressed as the matrix exponential of the transition rate matrix K_N of the promoter cycle (Eq. 7):

$$P_t(g|g') = \exp\left(K_N \Delta t\right)$$

The discrete waiting time distribution P_W is then given by:

$$P_W(n) = F_n(g = 0)$$

which is the probability of having observed the gene in the active state for the first time after n observations, or in term of time after $t = n\Delta t$, knowing that the system was initially in the first inactive state.

The discrete distribution of active periods can computed in a similar manner. Starting in $g = 0$ the active state, we defined an initial vector F_0 of size $N + 1$:

$$F_0(g) = \begin{cases} 1 & \text{if } g = 0 \\ 0 & \text{otherwise} \end{cases}$$

and the following recursion scheme:

$$F_{i+1}(g) = P_t(g|g' = 0)F_i(g' = 0)$$

The discrete waiting time distribution P_W is then given by:

$$P_W(n) = \sum_{g \neq 0} F_n(g)$$

where the sum runs over all promoter states except the active one $g = 0$.

6.3 Modeled steady-state distribution of mRNA

Although an analytical expression for the mRNA steady-state distribution of the promoter cycle was derived by Zhang et al. (Zhang *et al.* , 2012), this expression proved to be rather unpractical. Instead, we solved numerically the master equation (Eq. 6) at steady-state:

$$LP_s(mg) = 0$$

The steady-state distribution of mRNA is then given by the marginal distribution $P_s(m) = \sum_g P_s(mg)$.

6.4 Inferred vs modeled distributions

We first validated our approach, by performing Gibbs sampling on the toy examples presented in section 5.2. We then applied the method on the full set of clones, and compared the distributions predicted by the optimal model (modeled distributions) with the inferred ones.

Toy examples: Having already determined the optimal model for the four sets of simulated cells described in section 5.2 (Toy examples paragraph), we then performed Gibbs sampling using the optimal model as a prior to reconstruct the distributions of silent periods, active periods and mRNAs for each synthetic population. We also computed the modeled distributions from the optimal model according to sections 6.2 and 6.3. The resulting distributions are given in Figure S2A, S3A and S4A respectively. Both approaches lead to very similar results, displaying an excellent agreement, which was in fact expected in this case. Indeed, the analyzed population were homogenous (sharing the same kinetic parameters), therefore the optimal model should fully capture the stationary properties of the data.

Clones: We followed a similar approach than described in the previous paragraph, applying the method on the full set of clones, whose inferred kinetic parameter are listed in Table EV3. The resulting distributions, namely the silent period, the active period and mRNA steady-state distribution, are given in Figure S2B, S3B and S4B respectively. Regarding the silent periods (Figure S2B),

the modeled distribution matched fairly well the inferred one, confirming the previously observed signature of sequential inactive processes in the silent period of the genes (Suter *et al.* , 2011). Similarly, the modeled active periods (Figure S3B) are consistent with the inferred one. Although refractory periods in the active state cannot be ruled out, we did not observe peaked active periods in the analyzed clones. Since the average active periods were rather short compared to the sampling time Δt, it would have been difficult to detect the signature of sequential active states. Of note, we previously reported that *Ctgf* after serum stimulation exhibited a first longer lasting burst ($\tau_a \sim$ 30-40 min) whose duration was constrained and followed a peaked distribution (Molina *et al.* , 2013).

On the other hand, the inferred mRNA steady-state distributions (Figure S4B) can deviate from the modeled one. Inferred distributions tend to be wider, confirmed by the larger noise value $\sigma^2/\langle m\rangle^2$. Moreover, some clones (*NcKap1* and *Ctgf*) possess a peak at lower expression level reflecting substantial heterogeneity in mRNA expression in the cell population. Notably, *Dbp* shows considerable weight in its distribution at very low transcripts number (around 0 and 1 transcripts), resulting from long silent periods due to the circadian oscillations. We already showed evidence in a previous study (Molina *et al.* , 2013), that Gibbs sampling enables faithful reconstruction of the true dynamics, provided that data is strong enough compared to the prior (here the optimal model), consequently the observed features are unlikely to be artifacts of the method. Further evidence thereof is given in section 7.3.

7 Biological noise

The stochastic description of gene expression provides an explicit framework to connect the mechanisms involved in the expression process, although quite coarse-grained, with the observed fluctuations in number of proteins or mRNAs. Typically, those fluctuations can be characterized by defining dispersion measure on the steady-state distribution of molecules. The most common measures are the noise $\eta^2 = \frac{\sigma^2}{\mu^2}$ defined as the variance over the mean squared, and the noise strength, also called Fano factor, $F = \frac{\sigma^2}{\mu}$ which can be interpreted as a measure of deviation from the Poisson distribution.

An important step which is required in order to characterize the fluctuations of the system is to disentangle the different component of the measured noise, namely to separate the intrinsic contributions which are explicitly modeled from the extrinsic ones resulting from a fluctuating environment. In the following subsections, we first review analytical results regarding intrinsic transcriptional noise of the promoter cycle, then show how the noise propagates at the protein level and finally we present an approach to estimate the intrinsic and extrinsic component of the measured transcriptional noise.

7.1 Transcriptional noise of the promoter cycle

Here, we compute the noise of the promoter cycle by solving the equations for the mean and the covariance, derived from the master equation (Eq. 5), at steady-state (Lestas *et al.* , 2008). The mean of the species $\langle \mathbf{n} \rangle = (\langle m \rangle, \langle g_0 \rangle, ..., \langle g_{N-1} \rangle)^t$ is governed by the following differential equation:

$$\frac{d}{dt} \langle \mathbf{n} \rangle = f(\langle \mathbf{n} \rangle)$$

where $f(\langle \mathbf{n} \rangle) = \sum_i \mathbf{r}_i w_i(\langle \mathbf{n} \rangle)$ with $\mathbf{r}_i$ the state change vectors, w_i the propensity functions and the sum runs over all reaction channels. Using the fact that $\langle g_N \rangle = 1 - \sum_{k=0}^{N-1} \langle g_k \rangle$ and introducing the different timescales of the system as defined in Section 4.1, with $\tau_m = \gamma_m^{-1}$, we obtained:

$$f(\langle \mathbf{n} \rangle) = \begin{cases} k_m \langle g_0 \rangle - \langle m \rangle / \tau_m \\ (1 - \sum_{k=0}^{N-1} \langle g_k \rangle)/\tau_N - \langle g_0 \rangle / \tau_a \\ \langle g_0 \rangle / \tau_a - \langle g_1 \rangle / \tau_1 \\ \langle g_1 \rangle / \tau_1 - \langle g_2 \rangle / \tau_2 \\ \vdots \\ \langle g_{N-2} \rangle / \tau_{N-2} - \langle g_{N-1} \rangle \tau_{N-1} \end{cases}$$

since the propensity functions are linear functions of the species, $f(\langle \mathbf{n} \rangle) = A \langle \mathbf{n} \rangle + \mathbf{f}_0$ where A is the drift matrix. At steady-state, the mean of the species $\langle \mathbf{n} \rangle$ is obtained by solving the following linear system:

$$A \langle \mathbf{n} \rangle + \mathbf{f}_0 = 0 \tag{16}$$

The mean of the different species is then given by

$$\begin{aligned}
\langle m \rangle &= k_m \tau_m \frac{\tau_a}{\tau_a + T} = b \frac{\tau_m}{\tau_a + T} \\
\langle g_0 \rangle &= \frac{\tau_a}{\tau_a + T} \\
\langle g_1 \rangle &= \frac{\tau_1}{\tau_a + T} \\
&\vdots \\
\langle g_N \rangle &= \frac{\tau_N}{\tau_a + T}
\end{aligned}$$

Owing to the linearity of the propensity functions, the covariance of the species $\Sigma_{ij} = \langle \Delta n_i \Delta n_j \rangle$, with $\Delta n_i = n_i - \langle n_i \rangle$, is exactly governed by the following time-dependent Lyapunov equation (Lestas *et al.* , 2008):

$$\frac{d}{dt}\Sigma = A\Sigma + \Sigma A^{\mathrm{t}} + D \tag{17}$$

with D the diffusion matrix defined as $D = \sum_i \mathbf{r}_i w_i(\langle \mathbf{n} \rangle) \mathbf{r}_i^{\mathrm{t}}$ where, again, the sum runs over all reactions channels. By solving the Lyapunov equation at steady-state for increasing number of inactive states N, *i.e.*

$$A\Sigma + \Sigma A^{\mathrm{t}} + D = 0 \tag{18}$$

it can be shown recursively, after proper factorization of the different terms[1], that the variance on the mRNA copy number $\sigma^2 = \Sigma_{11}$ is exactly given by the following expression:

$$\sigma^2 = \langle m \rangle \left(1 + \langle m \rangle \eta_c^2\right)$$

where η_c^2 depends on the number of inactive states N and the timescales:

$$\eta_c^2 = \frac{(T - \tau_m) \displaystyle\prod_{i=1}^{N} (\tau_m + \tau_i) + \tau_m^{N+1}}{(\tau_m + \tau_a) \displaystyle\prod_{i=1}^{N} (\tau_m + \tau_i) - \tau_m^{N+1}} \tag{19}$$

[1]This tedious process was performed using Mathematica®

It is worth mentioning that the same expression for the variance was already obtained by Zhang et al. (Zhang *et al.* , 2012), namely they derived the analytical expression for the steady-state distribution of the promoter cycle and its moment generating functions, which enabled them to compute the aforementioned expression.

The intrinsic transcriptional noise $\eta^2 = \frac{\sigma^2}{\langle m \rangle^2}$ is then given by

$$\eta^2 = \frac{1}{\langle m \rangle} + \eta_c^2 = \eta_p^2 + \eta_c^2 \tag{20}$$

where η_p^2 is the Poisson contribution to the noise due to the stochastic production and degradation of mRNAs, and η_c^2 is the promoter noise due to random fluctuations in the promoter cycle. It was already clear that the noise contribution of the cycle η_c^2 could be lowered independently of the expression level (Pedraza & Paulsson, 2008), by increasing the number of inactive states, provided that the timescales $\{\tau_i\}$ are similar, and effectively reducing the variance of the total inactive time T. Moreover, the reduction is optimal if all the timescales of the inactive subprocess are equal (Zhang *et al.* , 2012), namely if $\tau_i = T/N \ \forall i$.

In order to acquire an intuitive understanding of the noise dependance on the promoter cycle parameters, we then aim to investigate various limiting regime. Without loss of generality, the two components of the transcriptional noise (Eq. 20) can be expressed in term of fundamental dimensionless quantities:

$$\eta_p^2 = \frac{f}{b}$$
$$\eta_c^2 = f \cdot C$$

where b is the burst size, f the normalized completion time of the cycle $f = (\tau_a + T)/\tau_m$ and C a factor which depends on the structure and the timescales of the cycle, *i.e.* $C(\tau_m, \tau_a, \{\tau_i\})$. The factor C is always positive and smaller or equal to 1. This property follows from the fact that, for fixed τ_m, τ_a and T, the promoter noise η_c^2 is always maximal for $N = 1$, namely $\eta^2_{c,(N=1)} \geq \eta^2_{c,(N>1)}$ (Zhang *et al.* , 2012), and consequently

$$C_{(N>1)} \leq C_{(N=1)} = \frac{\eta^2_{c,(N=1)}}{f} = \frac{T^2}{(\tau_a + T)^2 + \tau_a T (\tau_a + T)/\tau_m} \leq 1 \tag{21}$$

In a constitutive regime ($T \ll \tau_a$), C goes to zero, while in a bursting regime ($\tau_a \ll T \sim \tau_m$), C goes to one for a simple switch ($N = 1$).

The noise reduction provided by an increase in number of inactive states N is encoded in the factor C, namely C tends to decrease while N increases. This effect can be further characterized by assuming that the decay time of the transcripts τ_m is much larger than the timescales of the inactive subprocesses, i.e. $\tau_i \ll \tau_m \ \forall i$ or equivalently $T \ll \tau_m$. Formally, computing the limit of C

when τ_m goes to infinity, we obtain:

$$\lim_{\tau_m \to \infty} C(\tau_m, \tau_a, \{\tau_i\}) = \frac{T^2 - \sum_{i>j} \tau_i \tau_j}{(\tau_a + T)^2}$$

Further assuming that the timescales of the inactive subprocesses are equal, $\tau_i = T/N\ \forall i$, consequently getting a lower bound on C, leads to a simple expression:

$$C(\tau_a, T, N) = \frac{T^2}{(\tau_a + T)^2} \left(\frac{1}{2} + \frac{1}{2N} \right) \leq C(\tau_a, \{\tau_i\}) = \frac{T^2 - \sum_{i>j} \tau_i \tau_j}{(\tau_a + T)^2}$$

It follows that the noise reduction provided by N is optimal for $\tau_a \ll T$. Indeed, in the bursting limit, C solely depends on N:

$$C(\tau_a, T, N) \underbrace{\longrightarrow}_{\tau_a \ll T} C(N) = \frac{1}{2} \left(1 + \frac{1}{N} \right)$$

In that regime, the Fano factor only depends on the burst size and the number of inactive states:

$$F = 1 + \frac{\eta_c^2}{\eta_p^2} = 1 + b \cdot C = 1 + \frac{b}{2} \left(1 + \frac{1}{N} \right)$$

Similarly, the promoter noise takes a simple form:

$$\eta_c^2 = f \cdot C = \frac{T}{2\tau_m} \left(1 + \frac{1}{N} \right)$$

Interestingly, at fixed T/τ_m, the promoter noise η_c^2 decreases approximately as $1/N$, and at large N its contribution can be halved compared to a simple switch (N=1).

Although the assumptions used to derive the expression above might sound drastic, the approximation remains valid for the analyzed genes since they are bursting and the decay time $\tau_m \sim 100$ minutes is generally larger than the silent period T. In fact, the assumption that $T \ll \tau_m$ can be relaxed. Only assuming a bursting regime ($\tau_a \ll T$) and equal partition of the silent period ($\tau_i = T/N\ \forall i$), the limit of C for large N is given by

$$\lim_{N \to \infty} C(\tau_m, T, N) = \frac{(\alpha - 1)\exp(\alpha) + 1}{\alpha \exp(\alpha) - \alpha} = C(\alpha)$$

where $\alpha = \frac{T}{\tau_m}$. Of note, if $T \gg \tau_m$, the number of inactive steps N does not provide any noise reduction:

$$\lim_{\alpha \to \infty} C(\alpha) = 1$$

While for $T \ll \tau_m$, the noise reduction is optimal:

$$\lim_{\alpha \to 0} C(\alpha) = \frac{1}{2}$$

In the case where $T \sim \tau_m$, *i.e.* $\alpha \sim 1$, the maximal reduction provided by C is still fairly close to the one obtained in the optimal regime $T \ll \tau_m$. Indeed, $C(1) \simeq 0.58$, which only yields an error of 16% on the maximal attainable noise reduction.

7.2 Protein noise

So far, we focused on mRNA noise and here we will investigate how transcriptional noise propagates at the protein level and more precisely how this propagation is affected by the half-life of both the mRNA and the protein. The first question is especially relevant since a large proportion of biological processes are regulated by proteins. Consequently the protein noise should be determinant with regards to efficient regulation. The second question is of particular interest regarding our experimental setup. Recalling that the half-life of both the luminescent reporter ($\tau_{1/2} \sim 20$ min) and its mRNA ($\tau_{1/2} \sim$ 40-100 min) are artificially shorter than what is measured for endogenous mRNA and proteins (Sharova *et al.* , 2009; Schwanhäusser *et al.* , 2011), it is important to examine the effect of longer half-lives on the modeled noise.

Since in our model the proteins are governed by a simple birth-death process coupled through the effective translation rate with the mRNAs dynamics, the protein noise results from this one stage cascade. With good approximation (Pedraza & Paulsson, 2008), the protein noise is given by

$$\eta^2 \approx \frac{1}{\langle p \rangle} + \frac{\tau_m}{\tau_p + \tau_m} \eta_m^2 \tag{22}$$

with $\tau_p = \gamma_p^{-1}$. We recognized in the first term the Poisson contribution and the second one describes the time averaging or filtering of the mRNA noise. This expression would be exact if the transcripts were simply described by a birth-death process, and it remains a good approximation for the promoter cycle, as long as the decay time of the proteins τ_p and the transcripts τ_m are large compared to the timescales of the cycle τ_a and $\{\tau_i\}$.

By comparing the exact protein noise, computed numerically by solving the Lyapunov equation for the full system (Eq. 18), with the mRNA noise predicted by the optimal model for the different clones, we show in Figure S9A that the noise transmission closely follows the expression above (Eq. 22). Indeed, computing the noise using the experimentally measured decay time $\tau_p \sim 30$ min and $\tau_m \sim 100$ min (see Table EV1) and using more realistic values ($\tau_p = 5$ h and $\tau_m = 3$ h), in both cases the slope of the linear regression matches closely the factor $\tau_m/(\tau_p + \tau_m)$. Thus, the mRNA noise is transmitted linearly at the protein level, since the protein Poisson noise is negligible in practice, owing to the large protein copy number $\langle p \rangle > 100$ observed in most clones.

Having shown a simple linear relation between transcriptional and protein noise in our model,

we can already anticipate that the results related to noise reduction at the transcriptional level should remain valid at the protein level. Indeed, when estimating the noise reduction as a ratio of the noise of the one state model compared to the N states model, the prefactor $\tau_m/(\tau_p + \tau_m)$ drops. Moreover, the noise reduction offered by the N states model at the protein level is almost unaffected by the corrected values of τ_m and τ_p (Figure S9B).

7.3 Separation of extrinsic & intrinsic noise

One approach to disentangle the intrinsic noise from the extrinsic noise is based on the dual reporters setup (Swain *et al.* , 2002; Elowitz *et al.* , 2002), where two reporters of different color inserted in the same cell are driven by identical promoters. Measuring the correlation between the expression of the two reporters in a isogenic cells population allows estimation of the extrinsic component of the noise, namely fluctuations due to the environment (fluctuations in transcription factor copy number, polymerase, etc) affecting both genes equally. Unfortunately, adapting the dual reporters approach to our short-lived luciferase assay seems unrealistic for endogenous genes, since we would have to target both alleles of the randomly selected gene, which in itself would be extremely challenging, moreover both alleles are not necessarily expressed and identical. Therefore, we decided to split the noise based on the assumption that the environment is static, neglecting the extrinsic dynamics which cannot be deciphered without explicit treatment in the case of a single reporter. Such an approximation is satisfactory as long as the extrinsic processes are slow enough such that they can be considered constant during the recording period, otherwise the intrinsic contribution would be overestimated (Hilfinger & Paulsson, 2011).

Applying the law of total variance which splits the variance into the expectation of a conditional variance and the variance of a conditional expectation, we separated the total transcriptional variance of the cell population σ_m^2 in term of a variance $\sigma^2_{\langle m|i\rangle}$ coming from the environment of the different cells i, and an unexplained variance $\left\langle \sigma^2_{m|i} \right\rangle$:

$$\sigma_m^2 = \left\langle \sigma^2_{m|i} \right\rangle + \sigma^2_{\langle m|i\rangle} = \underbrace{\sum_{i=1}^{N_{cell}} w_i \sigma^2_{m|i}}_{\sigma^2} + \underbrace{\sum_{i=1}^{N_{cell}} w_i (m_i - \langle m \rangle)^2}_{\sigma_e^2} \tag{23}$$

where $\sigma^2_{m|i}$ is the transcriptional variance of ith cell, m_i its mean and $\langle m \rangle$ the mean of the population. The weights w_i account for unequal recording durations, and were chosen such that $w_i = L_i / \sum_{i=1}^{N_{cell}} L_i$ with L_i the length of the recording of ith cell. The unexplained variance can be related to the intrinsic contribution σ^2 and the explained one to the extrinsic contribution σ_e^2 (Hilfinger & Paulsson, 2011). Although the intrinsic contribution as defined above does not necessarily correspond to the intrinsic variance in absence of extrinsic noise (the mean variance is usually not equal to the modeled variance obtained with the mean kinetic parameters), the splitting still

provides a good approximation of intrinsic variability as demonstrated in simulated data below.

Validation: Having defined a splitting of the total variance in the context of a static environment, we tested whether we could potentially separate both components in data subject to extrinsic noise using Gibbs sampling (see Section 6.1). Moreover, we determined what fraction of intrinsic and extrinsic noise the optimal model captures in that condition.

For that purpose, we generated 24 populations of 48 heterogenous simulated cells (see Section 5.2) whose parameters were drawn from a multivariate distribution. We sampled the parameters of the cells as follows, we first set the mean expression of the population $\langle m \rangle = 10$, the mean active period $\langle \tau_a \rangle = 6$ min and the mean silent period $\langle T \rangle = 60$ min. We then imposed correlations between the different parameters: m and τ_a (r=0.4), τ_a and T (r=0.5), m and T (r=-0.4). In addition, we fixed the coefficient of variation (CV) of τ_a and T to 0.2, and defined the CV of m as a control parameter, varying from 0.3 to 0.9, in order to span a representative range of total noise. Finally, we sampled the different cells using a lognormal multivariate distribution for the three parameters, and set $k_m = m\gamma_m(\tau_a + T)/\tau_a$ with $\gamma_m = 0.01$ min^{-1}. Special care was taken to avoid lowly expressed cells ($m < 1$) and very short active period ($\tau_a < 1$). The number of inactive states was fixed to $N = 2$ with a silent partition close to symmetric.

On those heterogenous cell populations, we first applied our inference framework to determine the optimal model and its kinetic parameters, defined as the mode of the posterior distribution of N and the mean of the posterior distribution of k_m, τ_a and $\{\tau_i\}$. We then estimated the noise η_m^2 of the optimal model as the mean of its posterior distribution using equation (Eq. 20). Finally, we performed Gibbs sampling on all the cells individually using the optimal model as prior, to reconstruct the single-cell mRNA distributions and thus estimate the mean expression m_i and the variance σ_i^2 of each cell (Figure S5A). We verified that the single-cell Gibbs estimates recovered accurately the true single-cell mean and variance computed from the Gillespie mRNA time traces of the different cells (Figure S5B). Then, using equation (Eq. 23), we computed the intrinsic and extrinsic contributions of the population total noise, from the single-cell Gibbs sampling estimates and directly from data.

First, we verified that Gibbs sampling enables accurate reconstruction of the population mRNA distribution defined as the mixture of each single-cell distribution, and thus provides an excellent estimation of the total transcriptional noise in population with heterogenous kinetic parameters. The population distribution predicted by optimal model captures mainly the intrinsic variance (average single-cell variance in the data). Finally, we compared the population noise estimates provided by both Gibbs sampling and the optimal model in all the 24 heterogenous populations with varying amount of extrinsic noise (Figure S5C-D). Gibbs sampling consistently recovers the total and intrinsic noise in the true populations while the optimal model offers a good estimate of the intrinsic noise though it slightly overestimates the true intrinsic noise by $\approx 13\%$. Notably, both methods provide estimates that are rather insensitive to the amount of extrinsic noise in the data.

Thus, in the context of static environment, we showed that the optimal model captures mainly intrinsic noise and that Gibbs sampling estimates can be used to separate the total noise in term of intrinsic and extrinsic noise.

Clones: Here, we aimed at estimating the extrinsic component of the transcriptional noise in our set of clones. As we have seen in Figure S4B, the steady-state distribution of mRNA estimated via Gibbs sampling do not necessarily agree with the theoretical distribution predicted by the optimal model. There is often a significant excess of noise in the Gibbs estimates which is not captured by the model. Since we have shown in previous paragraph that the Gibbs estimates reflect total noise with good agreement and that the noise predicted by the optimal model is close to intrinsic noise, those deviations observed in the clones constitute strong evidences for extrinsic noise. In the clones, the optimal model recovers on average 56% of the total noise (Figure S4B).

Considering the deviations in term of noise estimates between the optimal model and Gibbs sampling, we then verified whether they could be explained in the context of static environment, namely if the model noise indeed reflect the estimated intrinsic component. We first separated the total transcriptional noise according to equation (Eq. 23) from Gibbs sampled single-cell distribution as explained in previous paragraph, the resulting intrinsic and extrinsic contributions to noise are shown in Figure 5B. The error bars correspond to parametric bootstrap confidence intervals (5 and 95 percentiles) obtained by fitting the mean m_i and the variance σ_i^2 of the different cells with a multivariate lognormal distribution. Since circadian clones (*Bmal1a*, *Bmal1b* and *Dbp*) are subject to clear dynamical extrinsic variability due to the circadian oscillations (Suter *et al.* , 2011), attempting to split the total noise of those clones based on static environment assumption would have led to underestimation of extrinsic noise. Therefore, we excluded them from this part of the analysis. Finally, we compared in Figure 5C the estimated intrinsic contribution to the optimal model prediction, on average the optimal model recovers 83% of the intrinsic noise.

8 Single-cell RNA-seq

To test the prediction of the promoter cycle model regarding biological noise for TATA and TATA-less promoters genome-wide, we aimed at inferring the noise from single-cell RNA-seq in mouse embryonic stem-cells (Grün *et al.* , 2014). We first modeled the measured noise in term of mRNA counts in function of the biological distribution of mRNA, the sensitivity of the technique and variability of the recovery rate across cells. We then use the derived expression of the measured noise to infer the biological noise over the range of expression covered by TATA and TATA-less genes.

8.1 Modeling single-cell RNA-seq

Single-cell RNA-seq combined with unique molecular identifier (UMI) enables quantification of the mean transcript abundance and the transcript variability genome-wide (Grün *et al.* , 2014). The technique can be modeled as binomial sampling of transcripts for each gene with a probability q corresponding to the sensitivity or recovery rate. Providing some variability on the sensitivity q among the cells, the probability to measure n transcripts for a given gene in a single-cell can be expressed in term of the true distribution of transcripts $P(m|\langle m\rangle, \sigma_m^2)$ and the distribution of the sensitivity $P(q|\langle q\rangle, \sigma_q^2)$ as follows:

$$P(n|\langle q\rangle, \sigma_q^2, \langle m\rangle, \sigma_m^2) = \int dq \sum_m P_b(n|m,q) P(m|\langle m\rangle, \sigma_m^2) P(q|\langle q\rangle, \sigma_q^2) \tag{24}$$

where $P_b(n|m,q)$ stands for the binomial distribution with m draws (true number of transcripts) and probability of success q. The first two central moments $\langle n\rangle$ and σ_n^2 can be directly calculated from expression (24) in term of the distribution parameters $(\langle q\rangle, \sigma_q^2, \langle m\rangle, \sigma_m^2)$ i.e. the mean and the variance of the sensitivity and the mRNA copy number. We then obtained:

$$\begin{aligned}
\langle n\rangle &= \langle q\rangle \langle m\rangle \\
\sigma_n^2 &= \left(\langle q\rangle - \langle q\rangle^2 - \sigma_q^2\right)\langle m\rangle + \left(\langle q\rangle^2 + \sigma_q^2\right)\sigma_m^2 + \langle m\rangle^2 \sigma_q^2
\end{aligned}$$

Thus, the measured noise η_n^2 can be expressed in term of the biological noise η_m^2 and the sensitivity noise η_q^2 as follows:

$$\eta_n^2 = \frac{\sigma_n^2}{\langle n\rangle^2} = \left(\frac{1}{\langle q\rangle} - \left(1+\eta_q^2\right)\right)\frac{1}{\langle m\rangle} + \left(1+\eta_q^2\right)\eta_m^2 + \eta_q^2 \tag{25}$$

The measured noise results from a combination of different contributions, namely the sampling noise that leads to Poisson noise $\left(\frac{1}{\langle q\rangle} - \left(1+\eta_q^2\right)\right)\frac{1}{\langle m\rangle}$, the sensitivity noise η_q^2 that set a lower bound on the measured noise for highly abundant transcripts, and the biological noise η_m^2 up to a factor

$\left(1+\eta_q^2\right)$.

8.2 Inference of biological noise genome-wide

In order to inferrer the average biological noise η_m^2 all along the range of expression covered by the genes, we first partitioned the measured noise η_n^2 in $K = 8$ bins S_k with mean number of counts $\langle n \rangle_k$, such that the partition covered the measured range of average counts with similar number of data points in each bin. We then fitted the expression for the measured noise η_n^2 (25) in function of the average number of counts $\langle n \rangle_k$ in the different bins. We assumed gaussian measurement noise (characterized by σ^2) on the log-value of η_n^2 (multiplicative noise) and expressed the likelihood of the measured η_n^2 in term of the parameters $\langle q \rangle, \eta_q^2, \eta_m^2$, as follows:

$$\mathcal{L}(D|\{\eta_m^2\}, \langle q \rangle, \eta_q^2, \sigma^2) = \prod_{k=1}^{K} \prod_{i=1}^{|S_k|} \mathcal{N}\left(y_i \in S_k | \mu_k = \log \eta_n^2(\langle n \rangle_k, \eta_m^2, \langle q \rangle, \eta_q^2), \sigma^2\right) \tag{26}$$

where y_i stands for the log-value of the measured noise η_n^2 of gene i belonging in the bin S_k with average number of counts $\langle n \rangle_k$ and $\mathcal{N}\left(y|\mu, \sigma^2\right)$ corresponds to the normal distribution with mean μ and variance σ^2. The sensitivity parameters $\langle q \rangle$ and η_q^2 were estimated from spike-in experiments, while the global parameter σ^2 and the biological noise η_m^2 for each bin were inferred as the mean of the posterior distribution estimated by MCMC sampling. Having inferred η_m^2 in function of $\langle n \rangle$, we finally converted the average number counts to mean mRNA copy number as $\langle m \rangle = \langle n \rangle / \langle q \rangle$.

Control The control data were obtained by pooling several thousands cells together and splitting the pool in single-cell equivalent (Grün *et al.* , 2014). Although such an approach reduce the biological variability to negligible level, the splitting introduces again Poisson noise for lowly to moderately abundant transcripts. Indeed, the splitting process can be modeled as multinomial sampling from the pool with probability f_g for each gene corresponding to the fraction of available transcripts. Since a large number of cells are pooled together, the fraction f_g is essentially given by $f_g = \frac{\langle m_g \rangle}{\sum_{g=1}^{G} \langle m_g \rangle}$ with $\langle m_g \rangle$ the average transcript abundance for gene g. Assuming that each cell contain on average M transcripts in total ($\sim 5 \cdot 10^5$ transcripts in mESC cells), the joint probability for transcript copy number of each gene n_g is then given by the following multinomial distribution:

$$P(n_1, ..., n_G | f_1, ..., f_G, M) = \frac{M!}{n_1! \cdot ... \cdot n_G!} f_1^{n_1} \cdot ... \cdot f_G^{n_G}$$

Since M is large, the joint distribution above can be well approximated by a product of independent binomial distributions, that can be further approximated to Poisson distributions due to small f_g:

$$P(n_1, ..., n_G | f_1, ..., f_G, M) \approx \prod_{g=1}^{G} P_b(n_g | f_g, M) \approx \prod_{g=1}^{G} P_p(n_g | \lambda_g = M f_g)$$

Thus, the number of transcripts in the control cells is subject to Poisson noise $\eta_m^2 = \frac{1}{\langle m \rangle}$. Variability on the total number transcripts per cell M would only add a constant term independent on the average expression $\langle m \rangle$.

We then performed inference of the biological noise η_m^2 in the control data (both 2i and serum condition) according to previous paragraph, using initially the value for $\langle q \rangle$ and η_q^2 estimated from the spike-in (3.4% and 0.05 respectively). We recover the predicted Poisson noise in the control, up to a multiplicative factor in expression, suggesting that the average sensitivity $\langle q \rangle$ is likely underestimated by the spike-in experiments. Indeed, it was mentioned that sensitivity estimation performed by single molecule RNA-FISH led to higher $\langle q \rangle$ (around 10%), the discrepancy was speculated to result from degradation of spike-in RNA or cellular RNA being more protected during cell lysis (Grün *et al.* , 2014). Thus, we estimated η_m^2 using $\langle q \rangle = 0.1$ that led to better agreement between the theoretical Poisson noise and the inferred curve (Figure 7C-D), although a systematic excess of noise remains that is not explained by the simple model.

Single cells In real cells, the biological noise results from a combination of intrinsic noise and extrinsic noise $\eta_m^2 = \eta^2 + \eta_e^2$. As we have seen previously, the intrinsic noise η^2 can be expressed in term of Poisson noise $\eta_p^2 = \frac{1}{\langle m \rangle}$ and promoter noise η_c^2 (Equation 20). Providing both alleles are transcribed and uncorrelated, the promoter noise would only contribute half as much compared to a single allele. Thus the modeled biological noise should behave as follows:

$$\eta_m^2 = \frac{1}{\langle m \rangle} + \frac{1}{2}\eta_c^2 + \eta_e^2 \tag{27}$$

According to our inference of promoter-cycle model from time-lapse measurement of single-allele driven luminescent reporter, we identified two groups of promoters: simple promoter with a single silent interval ($N = 1$) mainly associated with TATA box and more complex promoter cycle ($N > 1$) observed only in TATA-less promoters. Providing a significant fraction of genes are bursting, the promoter noise would be approximately given by $\eta_c^2 = \frac{f}{2}\left(1 + \frac{1}{N}\right)$ with $f = \frac{\tau_a + T}{\tau_m}$. Further assuming that the average mRNA life-time are roughly similar for TATA and TATA-less genes (Sharova *et al.* , 2009), TATA promoters owing to $N = 1$ should exhibit larger promoter noise than TATA-less promoters.

To test this hypothesis, we inferred the biological noise η_m^2 in single-cell data (both 2i and serum condition) for TATA and TATA-less promoters according to the EPD database (Dreos *et al.* , 2013). We found a significant enrichment for noise in TATA promoters compared to TATA-less promoters all along the range of average mRNA level (Figure 7C-D with $\langle q \rangle = 0.1$), though the difference lessen with expression. The latter effect could result from an increase of mRNA life-time τ_m and a lengthening of the active period τ_a for highly expressed transcripts. We verified whether the mRNA half-life correlates with expression, we did not find a significant trend or difference between TATA and TATA-less genes (Sharova *et al.* , 2009). Thus, the effect likely results from a lengthening of the

active period τ_a in both TATA and TATA-less genes at high expression. Moreover, the biological noise reaches a plateau at high expression suggesting substantial amount of extrinsic noise in mouse embryonic stem cells. Interestingly, the noise is overall larger in serum than 2i condition, consistent with the fact that serum condition leads to more heterogenous cell population. Finally, we tested wether the number of alleles would affect the inferred biological noise (reduction of promoter noise by a factor 2 for two alleles), by comparing the noise of genes on chromosome X (single allele) with the noise of autosomal genes in both conditions (Figure S8A-B). Although less significant, we still find a similar enrichment for noise on chromosome X as predicted by the simple model, thus supporting the hypothesis that the observed noise enrichment for TATA promoters is of intrinsic nature (promoter noise). Quantitatively, the noise difference between TATA and TATA-less promoter around 0.1-0.2 (Figure 7C-D) is in the range of the predicted value of $f/4$, providing $T \sim$ 1-2 hours and $\tau_m \sim$ 1-20 hours.

9 Supplementary References

References

Andrieu, Christophe, De Freitas, Nando, Doucet, Arnaud, & Jordan, Michael I. 2003. An introduction to MCMC for machine learning. *Machine learning*, **50**(1), 5–43.

Bel, Golan, Munsky, Brian, & Nemenman, Ilya. 2010. The simplicity of completion time distributions for common complex biochemical processes. *Physical Biology*, **7**(1), 016003.

Dreos, René, Ambrosini, Giovanna, Cavin Périer, Rouayda, & Bucher, Philipp. 2013. EPD and EPDnew, high-quality promoter resources in the next-generation sequencing era. *Nucleic Acids Research*, **41**(Database issue), D157–64.

Durbin, Richard. 1998. *Biological Sequence Analysis.* Probabilistic Models of Proteins and Nucleic Acids. Cambridge University Press.

Elowitz, Michael B, Levine, Arnold J, Siggia, Eric D, & Swain, Peter S. 2002. Stochastic gene expression in a single cell. *Science*, **297**(5584), 1183–1186.

Flegal, James M, & Jones, Galin L. 2010. Batch means and spectral variance estimators in Markov chain Monte Carlo. *The Annals of Statistics*, **38**(2), 1034–1070.

Gelfand, Alan E, & Smith, Adrian F M. 1990. Sampling-Based Approaches to Calculating Marginal Densities. *Journal of the American Statistical Association*, **85**(410), 398–409.

Gillespie, Daniel T. 1977. Exact stochastic simulation of coupled chemical reactions. *The journal of physical chemistry*, **81**(25), 2340–2361.

Green, Peter J, & Hastie, David I. 2009. Reversible jump MCMC. *Genetics*, **155**(3), 1391–1403.

Grün, Dominic, Kester, Lennart, & van Oudenaarden, Alexander. 2014. Validation of noise models for single-cell transcriptomics. *Nature Methods*, **11**(6), 637–640.

Harper, Claire V, Finkenstädt, Bärbel, Woodcock, Dan J, Friedrichsen, Sönke, Semprini, Sabrina, Ashall, Louise, Spiller, David G, Mullins, John J, Rand, David A, Davis, Julian R E, & White, Michael R H. 2011. Dynamic analysis of stochastic transcription cycles. *PLoS Biology*, **9**(4), e1000607.

Hilfinger, Andreas, & Paulsson, Johan. 2011. Separating intrinsic from extrinsic fluctuations in dynamic biological systems. *Proceedings of the National Academy of Sciences*, **108**(29), 12167–12172.

Kemeny, John G, & Snell, James Laurie. 1976. *Finite markov chains.* Vol. 210.

Lestas, Ioannis, Paulsson, Johan, Ross, Nicholas E, & Vinnicombe, Glenn. 2008. Noise in Gene Regulatory Networks. *Automatic Control, IEEE Transactions on*, **53**, 189–200.

Molina, Nacho, Suter, David M, Cannavo, Rosamaria, Zoller, Benjamin, Gotic, Ivana, & Naef, Felix. 2013. Stimulus-induced modulation of transcriptional bursting in a single mammalian gene. *Proceedings of the National Academy of Sciences*, **110**(51), 20563–20568.

Munsky, Brian, & Khammash, Mustafa. 2006. The finite state projection algorithm for the solution of the chemical master equation. *The Journal of Chemical Physics*, **124**(4), 044104.

Peccoud, Jean, & Ycart, Bernard. 1995. Markovian modeling of gene-product synthesis. *Theoretical Population Biology*, **48**(2), 222–234.

Pedraza, Juan M, & Paulsson, Johan. 2008. Effects of molecular memory and bursting on fluctuations in gene expression. *Science*, **319**(5861), 339–343.

Rabiner, Lawrence R. 1989. A tutorial on hidden Markov models and selected applications in speech recognition. *Pages 257–286 of: Proceedings of the IEEE*. IEEE.

Schwanhäusser, Björn, Busse, Dorothea, Li, Na, Dittmar, Gunnar, Schuchhardt, Johannes, Wolf, Jana, Chen, Wei, & Selbach, Matthias. 2011. Global quantification of mammalian gene expression control. *Nature*, **473**(7347), 337–342.

Sharova, Lioudmila V, Sharov, Alexei A, Nedorezov, Timur, Piao, Yulan, Shaik, Nabeebi, & Ko, Minoru S H. 2009. Database for mRNA half-life of 19 977 genes obtained by DNA microarray analysis of pluripotent and differentiating mouse embryonic stem cells. *DNA research : an international journal for rapid publication of reports on genes and genomes*, **16**(1), 45–58.

Sidje, Robert B. 1998. Expokit: a software package for computing matrix exponentials. *ACM Transactions on Mathematical Software (TOMS)*, **24**(1), 130–156.

Suter, David M, Molina, Nacho, Gatfield, David, Schneider, Kim, Schibler, Ueli, & Naef, Felix. 2011. Mammalian genes are transcribed with widely different bursting kinetics. *Science*, **332**(6028), 472–474.

Swain, Peter S, Elowitz, Michael B, & Siggia, Eric D. 2002. Intrinsic and extrinsic contributions to stochasticity in gene expression. *Proceedings of the National Academy of Sciences of the United States of America*, **99**(20), 12795–12800.

Zhang, Jiajun, Chen, Luonan, & Zhou, Tianshou. 2012. Analytical distribution and tunability of noise in a model of promoter progress. *Biophysical Journal*, **102**(6), 1247–1257.

Zopf, C J, Quinn, Katie, Zeidman, Joshua, & Maheshri, Narendra. 2013. Cell-cycle dependence of transcription dominates noise in gene expression. *PLoS Computational Biology*, **9**(7), e1003161.
