# Supplementary material for: Structure of silent transcription intervals and noise characteristics of mammalian genes
Source: Mol Syst Biol. 2015 Jul 27;11(7):823. doi: 10.15252/msb.20156257 (PMC4547851; doi:10.15252/msb.20156257)
Supplement: Supplementary file 2 [file msb0011-0823-sd2.zip › Readme_TableEV1.rtf]

Table EV1: Measured translation rates k_p, degradation rates for both the protein y_p and the mRNA y_m, and number of analyzed cells for each clone.
