# Supplementary material for: Structure of silent transcription intervals and noise characteristics of mammalian genes
Source: Mol Syst Biol. 2015 Jul 27;11(7):823. doi: 10.15252/msb.20156257 (PMC4547851; doi:10.15252/msb.20156257)
Supplement: Supplementary file 4 [file msb0011-0823-sd4.zip › Readme_TableEV3.rtf]

Table EV3: Number of inactive states, kinetic parameters and partition of the silent period inferred with RJ-MCMC for the different clones. The parameters are computed as the mean of the posterior distribution and the errors as the 5th and 95th percentiles of the posterior distribution. In addition, we computed for the main parameters the Monte-Carlo relative standard error. For the partition of the silent period, we also computed the corrected intervals since the mean is biased on the restricted simplex (cf. Appendix Section 6.1). 
