# Supplementary material for: Structure of silent transcription intervals and noise characteristics of mammalian genes
Source: Mol Syst Biol. 2015 Jul 27;11(7):823. doi: 10.15252/msb.20156257 (PMC4547851; doi:10.15252/msb.20156257)
Supplement: Supplementary file 6 [file msb0011-0823-sd6.zip › Readme_scRNAseq.rtf]

Script_scRNAseq: Matlab script provided to reproduce the Figure 7 of the manuscript, from the data (Grün et al. 2014). The dataset GSE54695_data_transcript_counts.txt can be downloaded at http://www.ncbi.nlm.nih.gov/geo/query/acc.cgi?acc=GSE54695.Noise_scRNAseq.m: Main script reproducing the figuresLoglikelihood.m: Loglikelihood function called by the main script.Run_MCMC.m: MCMC sampler called by the main script.tata.txt: list of tata-box containing genes extracted from the EPDnew database (http://epd.vital-it.ch/EPDnew_select.php).chromX.txt: list of chromosome X genes.
