# Supplementary material for: Structure of silent transcription intervals and noise characteristics of mammalian genes
Source: Mol Syst Biol. 2015 Jul 27;11(7):823. doi: 10.15252/msb.20156257 (PMC4547851; doi:10.15252/msb.20156257)
Supplement: Supplementary file 7 [file msb0011-0823-sd7.pdf]

molecular systems biology

Structure of silent transcription intervals and noise characteristics of mammalian genes

Benjamin Zoller, Damien Nicolas, Nacho Molina, Felix Naef

Corresponding author: Felix Naef, Ecole Polytechnique Federale de Lausanne EPFL

Review timeline:

Submission date:	27 April 2015
Editorial Decision:	08 June 2015
Revision received:	23 June 2015
Editorial Decision:	29 June 2015
Acceptance letter:	3 July 2015

Editor: Thomas Lemberger

Transaction Report:

(Note: With the exception of the correction of typographical or spelling errors that could be a source of ambiguity, letters and reports are not edited. The original formatting of letters and referee reports may not be reflected in this compilation.)

1st Editorial Decision 08 June 2015

Thank you again for submitting your work to Molecular Systems Biology. We have now heard back from two of the three referees who accepted to evaluate the study. In view of the similar recommendations provided by the two reviewers, I prefer to make a decision now with the available reports rather than delaying further the process. As you will see, the present referees find the topic of your study of potential interest. They make a few constructive suggestions for modifications, which we would ask you to carefully address in a revision of the present work.

In view of the quantitative nature of the study, we would be particularly grateful if you could make sure that new experimental data is included to the submission as "Dataset". If reasonable and only when appropriate, it might also be a good idea to provide the code for the computational analysis and/or use literate programming tools to describe the analysis. This might be relevant for the analysis of the single cell RNA analysis.

Reviewer #2:

In this manuscript by Zoller et al., the authors use computational modeling to infer transcriptional noise encoded by the promoter. They analyze two data sets: 1) the single cell luciferase data published previously in the lab (Suter et al., Science, 2011) and 2) single-cell RNA seq data from ESCs (Grun et al., 2014). They specifically address the idea of a promoter 'refractory' period, which is a period in between transcription bursts when the gene cannot turn on again. The physical manifestation of this refractory period is a peaked distribution in off times instead of an exponential distribution. They model this refractory period as arising from a promoter which progresses through sequential states before activation. As the number of states increases (assuming that the states have equal lifetimes), the promoter becomes more regular and in fact can even be seen to oscillate, as has been described in other publications. The observation that I found most interesting is that burst size depends primarily on the

© European Molecular Biology Organization

promoter (as opposed to chromatin). Related to this observation, TATA promoters seem to be relatively simple, relying on 1 or 2 states before activation, whereas as the majority of promoters cycle through multiple (~ 7 states) before activation. These latter genes display less noise. The single cell data is stronger in my opinion than the RNA-seq data. There are a few limitations of this study (described below), but overall I found it rigorous, fairly easy to read and interesting. I recommend publication after some minor revisions.
1)One limitation is that promoter kinetics are inferred from protein time series. Although the authors acknowledge this caveat, this inference should be taken into account when comparing to studies which image RNA directly. Ultimately, there are many more assumptions involved in arriving at the actual promoter cycles.
2)Have the authors looked for any refractory periods in the on-state? Can they rule them out?
3)Are there other phenomena which can lead to a refractory period? For example if there are bursts of translation or bursts of RNA export, can these kinetic processes show up as a refractory period in the off state?
4)Fig. 3 D. I am a little unclear on what is being depicted here. Is the fact that the alternating dark and light grey bars have varying lengths for a single gene meant to indicate the variability? Would these bars be equivalent to Monte Carlo traces derived from their simulation parameters?

Reviewer #3:

In this manuscript Zoller et al. demonstrate that a model of multiple sequential metastable states of a promoter between bouts of transcriptional activation best explains time-course expression data in mammalian cells. By measuring both durations of these silent intervals and expression noise, they were able to parameterize a set of related minimal models that differed in the number of these metastable states, use a model-selection technique to choose between them, and discover two classes of genes - one with transitions between around 6 silent states and one with only 1 or 2 transcriptionally inactive states. Longer cycles should theoretically reduce transcriptional noise and their experimental data was consistent with this. Furthermore, TATA-containing promoters, known to be more variable on average than non-TATA ones, fell in the category with the simpler promoter cycle. They then looked at single-cell, single-allele RNA-seq data, estimated intrinsic expression noise, and found that TATA-containing genes had higher intrinsic noise than non-TATA genes.

At its core, this paper implements a simple model for gene activation that consists of a single active state followed sequential inactive states that produce a refractory period during which the gene becomes once again ready to be activated. They collect longitudinal data using a short-lived luciferase reporter encoded by a short-lived mRNA which gives them nicely time-resolved timecourses. They examine several promoter constructs and cell-lines where an endogenous promoter drives transcription. Then they estimate the likelihoods of their data given the models, and estimate parameters and select between models in a Bayesian framework. This all seems very well done. They construct simulated datasets, mimicking the experimental conditions, with varied parameters to test their analysis and to provide insight into interpreting the real data.

Although they state (page 4) that they will characterize the rate limiting steps of promoter progression, they deliver characterization of the dynamics of this but not the molecular details. Given the dynamics and timescales involved, they exclude certain molecular mechanisms behind the promoter cycles (like TF-DNA interactions) while arguing that histone modifications are more consistent with the dynamics and exploring this in the discussion. I do not mean to suggest that the authors need to nail down the molecular details in this paper, but their language there and in the abstract is a little vague on what they mean (e.g. "resolve" and "characterize") and could lead the reader to expect more than they provide.

The weakest part of the paper is the single-cell RNA-seq part. This is mostly from the nature of single-cell RNA-seq experiments (the data they use is from another paper). These experiments are noisy. Only a fraction of the RNA is recovered, there can be lots of biases - it's a hard experiment. The authors do a good job of laying out how they modeled single-cell RNA-seq to account for the sampling noise and sensitivity, and extract intrisinc noise. Their approach appears reasonable, and they do pull out a subtle difference between TATA-containing and TATA-less genes in mRNA noise that shows up in the cell-based data and not in a data derived from a homogenized multi-cell pool. In figure 7A&B they plot the data for all the genes and it can be seen that the TATA ones are largely contained within the TATA-less cloud, albeit typically at the slightly higher measured noise values. To get figures 7C&D, they bin the single-gene data estimate average mRNA noise within a bin. Their error bars are for this mean noise

level. While it is clear that these mean levels are different across a range of mean mRNA numbers, given the highly overlapping clouds of 7A&B it isn't clear to me that this would have much predictive or explanatory value for any particular gene.

All in all, this seems like a solid paper that will be of interest to a fairly sizable community interested in the process of transcription - both in dynamics and noise and in mechanism. There are a few minor corrections that should be made:

1) There are lots of grammatical errors, incorrect word choices, and missing words. I would highly recommend the authors ask a native English speaker to read and edit the manuscript.

2) The colors of the clones are hard to distinguish in the figures. Perhaps using different symbols for the similar colors would help.

3) It would be nice if the TATA vs. TATA-less genes could be distinguished in one of the figures. Perhaps 3D would be an appropriate place.

1st Revision - authors' response 23 June 2015

Reviewer #2:

In this manuscript by Zoller et al., the authors use computational modeling to infer transcriptional noise encoded by the promoter. They analyze two data sets: 1) the single cell luciferase data published previously in the lab (Suter et al., Science, 2011) and 2) single-cell RNA seq data from ESCs (Grun et al., 2014). They specifically address the idea of a promoter 'refractory' period, which is a period in between transcription bursts when the gene cannot turn on again. The physical manifestation of this refractory period is a peaked distribution in off times instead of an exponential distribution. They model this refractory period as arising from a promoter which progresses through sequential states before activation. As the number of states increases (assuming that the states have equal lifetimes), the promoter becomes more regular and in fact can even be seen to oscillate, as has been described in other publications. The observation that I found most interesting is that burst size depends primarily on the promoter (as opposed to chromatin). Related to this observation, TATA promoters seem to be relatively simple, relying on 1 or 2 states before activation, whereas as the majority of promoters cycle through multiple (~ 7 states) before activation. These latter genes display less noise. The single cell data is stronger in my opinion than the RNA-seq data. There are a few limitations of this study (described below), but overall I found it rigorous, fairly easy to read and interesting. I recommend publication after some minor revisions.

We thank this reviewer for his positive evaluation.

1) One limitation is that promoter kinetics are inferred from protein time series. Although the authors acknowledge this caveat, this inference should be taken into account when comparing to studies which image RNA directly. Ultimately, there are many more assumptions involved in arriving at the actual promoter cycles.

We certainly agree that inferring promoter kinetics by means of destabilized reporters implies several additional assumptions compared to direct measurements such as smRNA-FISH or MS2/PP7 imaging. Each method has their pros and cons. What we like about the destabilized luciferase is the sensitivity (~20 protein), low background, and ability to perform long-term time-lapse recordings (2-3 days). As we discussed, the temporal axis is key to resolve promoter kinetics, however, this inevitably requires that we specify a model. Moreover, this model needs to be parsimonious to allow inference, while sufficiently flexible to capture the key biological processes underlying gene expression.

We have now significantly re-written the Discussion section “Modeling promoter cycles” on p.17-18 to state the limitations and all assumptions more clearly. In particular, for what concerns the promoter cycles, we now discuss that our models assume discrete promoter states, and an ordered sequence of irreversible transitions leading to a transcriptionally active state.

2) Have the authors looked for any refractory periods in the on-state? Can they rule them out?

This is a good point. We agree that, in principle, on-times could be subject to refractory periods as well. To look into this, we compared the on-time distribution reconstructed by Gibbs sampling with the predicted exponential distribution for a single rate-limiting step (see new Figure S3). The property that both distributions closely match indicates that we do not observe a refractory period in the active state on the measured time scale of 5 minutes. Thus, we cannot rule out a short refractory period in the 'on' state in the order of seconds to one minute, but such periods would anyway be much shorter than the refractory periods in the 'off' state. Of note, under stimulated conditions where 'on' intervals can be longer, we previously reported a refractory 'on' period for the *Ctgf* gene (Molina et al., 2013).

We now discussed this and added Fig. S3 on p.10, top of the page.

3) Are there other phenomena which can lead to a refractory period? For example if there are bursts of translation or bursts of RNA export, can these kinetic processes show up as a refractory period in the off state?

Blocking transcription with actinomycin D immediately abolished bursting, which led us to conclude that the recorded bursts were of transcriptional origin (Suter et al., 2011). Therefore, it is unlikely that hypothetical bursts of RNA export would cause the refractory period. Moreover, globally controlled bursts of mRNA export could hardly account for the promoter specific kinetics we observed for the different clones.

4) Fig. 3D. I am a little unclear on what is being depicted here. Is the fact that the alternating dark and light grey bars have varying lengths for a single gene meant to indicate the variability? Would these bars be equivalent to Monte Carlo traces derived from their simulation parameters?

We agree that the caption of Figure 3D was not sufficiently detailed, and thus, the interpretation of the figure lacked clarity. We now improved this caption. Briefly, in Fig. 3D, we represented the partition of the silent period into the subintervals predicted by the optimal cycle model. In the figure the length of the intervals represents the estimate timescale of each subinterval. Since the different timescales are not necessarily equal in our model, the partition of the silent period can be asymmetric. The alternating dark and light grey were only used to facilitate the reading.

Reviewer #3:

In this manuscript Zoller et al. demonstrate that a model of multiple sequential metastable states of a promoter between bouts of transcriptional activation best explains time-course expression data in mammalian cells. By measuring both durations of these silent intervals and expression noise, they were able to parameterize a set of related minimal models that differed in the number of these metastable states, use a model-selection technique to choose between them, and discover two classes of genes - one with transitions between around 6 silent states and one with only 1 or 2 transcriptionally inactive states. Longer cycles should theoretically reduce transcriptional noise and their experimental data was consistent with this. Furthermore, TATA-containing promoters, known to be more variable on average than non-TATA ones, fell in the category with the simpler promoter cycle. They then looked at single-cell, single-allele RNA-seq data, estimated intrinsic expression noise, and found that TATA-containing genes had higher intrinsic noise than non-TATA genes.

At its core, this paper implements a simple model for gene activation that consists of a single active state followed sequential inactive states that produce a refractory period during which the gene becomes once again ready to be activated. They collect longitudinal data using a short-lived luciferase reporter encoded by a short-lived mRNA which gives them nicely time-resolved timecourses. They examine several promoter constructs and cell-lines where an endogenous promoter drives transcription. Then they estimate the likelihoods of their data given the models, and estimate parameters and select between models in a Bayesian framework. This all seems very well done. They construct simulated datasets, mimicking the experimental conditions, with varied parameters to test their analysis and to provide insight into interpreting the real data.

Although they state (page 4) that they will characterize the rate limiting steps of promoter progression, they deliver characterization of the dynamics of this but not the molecular details. Given the dynamics and timescales involved, they exclude certain molecular mechanisms behind the promoter cycles (like TF-DNA interactions) while arguing that histone modifications are more consistent with the dynamics and exploring this in the discussion. I do not mean to suggest that the authors need to nail down the molecular details in this paper, but their language there and in the abstract is a little vague on what they mean (e.g. "resolve" and "characterize") and could lead the reader to expect more than they provide.

Thank you for pointing this out, we fully agree with this important remark. We indeed need to be unambiguous that while we estimate the number and timescales of the rate limiting steps, we are not dissecting their molecular underpinning. This would be fantastic, but is unfortunately extremely difficult experimentally. We have thus adopted a clear terminology to avoid possible misinterpretations.

In the abstract, we now write: "Here, we use single allele time-lapse recordings in mouse cells to identify minimal models of promoter cycles, which inform on the number and durations of rate-limiting steps responsible for refractory periods.", and we used similar wording elsewhere in the text (p.4, bottom; p.17 top; p.22, top).

The weakest part of the paper is the single-cell RNA-seq part. This is mostly from the nature of single-cell RNA-seq experiments (the data they use is from another paper). These experiments are noisy. Only a fraction of the RNA is recovered, there can be lots of biases - it's a hard experiment. The authors do a good job of laying out how they modeled single-cell RNA-seq to account for the sampling noise and sensitivity, and extract intrisinc noise. Their approach appears reasonable, and they do pull out a subtle difference between TATA-containing and TATA-less genes in mRNA noise that shows up in the cell-based data and not in a data derived from a homogenized multi-cell pool. In figure 7A&B they plot the data for all the genes and it can be seen that the TATA ones are largely contained within the TATA-less cloud, albeit typically at the slightly higher measured noise values. To get figures 7C&D, they bin the single-gene data estimate average mRNA noise within a bin. Their error bars are for this mean noise level. While it is clear that these mean levels are different across a range of mean mRNA numbers, given the highly overlapping clouds of 7A&B it isn't clear to me that this would have much predictive or explanatory value for any particular gene.

We agree that, despite enormous progress, RNA-seq data are still noisy (though we found that the Grün data, by using the UMIs, is particularly suited for quantitative analyses). In fact, because of the caveats mentioned by the reviewer, we were positively surprised that signatures of promoter fluctuations could be detectable. As the referee acknowledges, we cannot make predictions on individual genes, but groups of genes can be separated. This is now stated more clearly in the text (p15). We did explore alternative explanations for the observed shift (see Discussion), but a difference in intrinsic promoter noise remains our best explanation, also because comparing genes on the X and autosomal chromosomes exhibits similar shift. Thus, we believe that the RNA-seq analysis provides a valuable complement the time-lapse analysis, since it uses a radically orthogonal approach and essentially confirms what we predicted from a limited number of single cell clones.

All in all, this seems like a solid paper that will be of interest to a fairly sizable community interested in the process of transcription - both in dynamics and noise and in mechanism.

We appreciate that this review found our paper robust and interesting.

There are a few minor corrections that should be made:

1) There are lots of grammatical errors, incorrect word choices, and missing words. I would highly recommend the authors ask a native English speaker to read and edit the manuscript.

Indeed, we have now asked a native English speaker in the group, Jake Yeung, to correct the language. We have corrected grammatical errors in the revised manuscript and the text reads much better now.

© European Molecular Biology Organization

2) The colors of the clones are hard to distinguish in the figures. Perhaps using different symbols for the similar colors would help.

Thanks for this. We fully agree, and have now added symbols in addition to the colors, which helps a lot.

3) It would be nice if the TATA vs. TATA-less genes could be distinguished in one of the figures. Perhaps 3D would be an appropriate place.

This is a good idea. We added this to Figure 3D.

2nd Editorial Decision 29 June 2015

Thank you again for submitting your work to Molecular Systems Biology. We are now satisfied with the modifications made and we will be able to accept formally your study for publication pending some minor amendments regarding the supplementary information formatting.

Revision - authors' response 3 July 2015

We are very pleased that our manuscript is accepted in MSB, we have now made all the change compatible with the new format.

Acceptance letter 3 July 2015

Thank you again for sending us your revised manuscript. We are now satisfied with the modifications made and I am pleased to inform you that your paper has been accepted for publication.
