# Supplementary material for: Structure of silent transcription intervals and noise characteristics of mammalian genes
Source: Mol Syst Biol. 2015 Jul 27;11(7):823. doi: 10.15252/msb.20156257 (PMC4547851; doi:10.15252/msb.20156257)
Supplement: Supplementary file 5 [file msb0011-0823-sd5.zip › Readme_Dataset_1.rtf]

Dataset 1: Set of luminescence time traces for each clone analyzed in the paper. The dataset includes former data from Suter et al. 2011 and the newly generated time traces for the H1 construct.
Individual signal files (*.signal) for each clone correspond to z-score, namely background subtracted signal divided by the standard deviation of the background.
For each clone, a background file (*.background) is provided and contains the mean background value and the standard deviation in grey level for each trace. In addition, the initial length of the movie and the final length after preprocessing of each trace is provided. Briefly, we removed initial and final sections of the recordings to avoid bias due to the thermalization of the microscope and dying cells at end of the movies. For additional details regarding the preprocessing, see Suter et al. 2011.
